# QSAR, Antimicrobial, and Antiproliferative Study of (*R*/*S*)-2-Thioxo-3,4-dihydropyrimidine-5-carboxanilides

**DOI:** 10.1021/acsomega.4c09899

**Published:** 2025-02-10

**Authors:** Mehul P. Parmar, Anwesha Das, Disha P. Vala, Savan S. Bhalodiya, Chirag D. Patel, Shana Balachandran, Nagesh Kumar Kandukuri, Shreya Kashyap, Adam N. Khan, Aday González-Bakker, Madan Kumar Arumugam, José M. Padrón, Arijit Nandi, Sourav Banerjee, Hitendra M. Patel

**Affiliations:** † Department of Chemistry, Sardar Patel University, Vallabh Vidyanagar, Gujarat 388120, India; ‡ Department of Pharmacy, Sanaka Educational Trust Group of Institutions (SETGOI), Malandighi, Durgapur, West Bengal 713212, India; § Cancer Biology Lab, Center for Molecular and Nanomedical Sciences, Sathyabama Institute of Science and Technology, Chennai, Tamil Nadu 600119, India; ∥ YMC Application Lab, Plot No. 78/A/6, Phase VI, Industrial Park Jeedimetla, Gajularamaram Village, Quthbullapur, Medchal, Hyderabad, Telangana 500055, India; ⊥ Division of Cancer Research, School of Medicine, University of Dundee, Dundee DD1 9SY, U.K.; # BioLab, Instituto Universitario de Bio-Orgánica Antonio González, Universidad de La Laguna, Avda. Astrofísico Francisco Sánchez 2, La Laguna 38206, Spain; ¶ Institute for Molecular Bioscience, The University of Queensland, 306 Carmody RoadSt Lucia Qld, Brisbane 4072, Australia

## Abstract

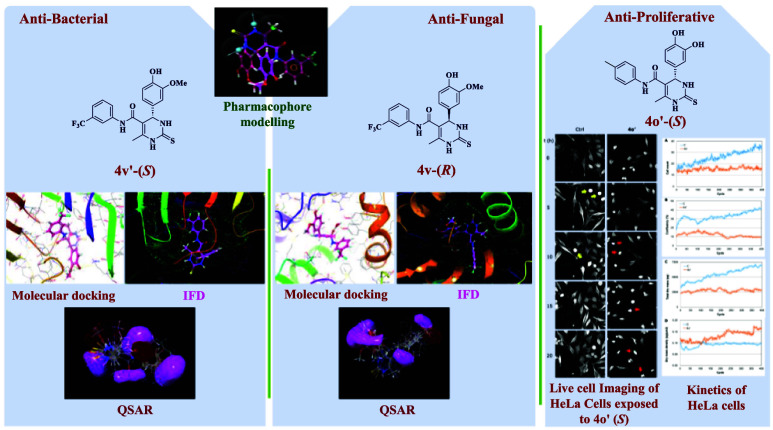

Owing to the significant contribution of three-dimensional (3D) field-based QSAR toward hit optimization and accurately predicting the activities of small molecules, herein, the 3D-QSAR, in vitro antimicrobial, molecular docking, and pharmacophore modeling studies of all the isolated (*R*/*S*)-2-thioxo-DHPM-5-carboxanilides exhibiting antimicrobial activity were carried out. The screening process was performed using 46 compounds, and the best-scoring model with the top statistical values was considered for bacterial and fungal targets *Bacillus subtilis* and *Candida albicans*. As a result of 3D-QSAR analysis, compound **4v**-(*S*)- and **4v**-(*R*)-isomers were found to be more potent compared to the standard drugs tetracycline and fluconazole, respectively. Furthermore, the enantiomerically pure isomers **4q**, **4d′**, **4n**, **4f′**, **4v**, **4q′**, **4c**, and **4p′** were found to be more potent than tetracycline and fluconazole to inhibit the bacterial and fungal growth against *B. subtilis*, *Salinivibrio proteolyticus*, *C. albicans*, and *Aspergillus niger*, respectively. Molecular docking analysis shows that with the glide score of −10.261 kcal/mol, **4v**-(*R*)-isomer was found to be more potent against the fungal target *C. albicans* and may target the 14-α demethylase than fluconazole. Furthermore, all compounds’ antiproliferative activity results showed that 4**o′** exhibited GI50 values between 8.8 and 34 μM against six solid tumor cell lines. Following the greater potential of **4o′** toward the *HeLa* cell line, its kinetics study and live cell imaging were carried out. These outcomes highlight the acceptance and safety as well as the potential of compounds as effective antiproliferative and antifungal agents.

## 1 Introduction

In recent years, experimental biological studies have become increasingly expensive, laborious, and time-consuming. Along with this, the in silico (computational) approaches may present a more cost-effective and dependable risk assessment technique.^1^ The three-dimensional (3D) quantitative structure–activity relationship (QSAR) screening method has been proven to be effective in driving hit optimization and precisely projecting the activities of possible small molecules compared to other computational methodologies.^2^ Wherein, the Hammett function and partition coefficient would become crucial in determining the relationship between structure and activity in which Hans and colleagues’ fundamental work on QSAR.^3^ This approach has been extensively utilized in the fields of drug development, pharmacy, and toxicology where it can provide an instant solution to fulfill the absent or limited experimental information.^1^ In recent years, molecular docking has been extensively utilized in academia and industrial areas as a straightforward and inexpensive technique for the prediction of the binding affinity of ligands to receptor proteins.^4,5^ Molecular docking analyzes the pose (orientation and confirmation) of molecules into the targeted protein’s binding site, which has been described using a scoring function.^6^ The number of cases of opportunistic fungal diseases such as *Candida albicans* has increased due to some advancements in modern medicine. Clinical medications aimed at treating fungal diseases mostly target CYP51, a cytochrome P450 enzyme necessary for the formation of sterols in eukaryotic cells. Therefore, we have chosen the crystal structure of sterol 14α demethylase (CYP51) from *C. albicans* in complex with the tetrazole-based antifungal drug candidate VT1161 (VT1) and *Bacillus subtilis* PabB for QSAR and MD studies against the fungal and bacterial strains, respectively.

The FPPL (First Fungal Priority List) published by the WHO report aims to raise awareness about the current and future impact of specific fungal diseases to drive global research.^7,8^ The results of this research reveal a detailed overview of issues associated with diagnosing, treating, and pursuing research and development goals related to fungal diseases. It also lists fungal pathogens into three categories: medium, high, and critical. According to this, *C. albicans* and *Aspergillus fumigatus* ranked 13^th^ and 14^th^ and public health ranked second and first in fungal pathogens, respectively. As per Denning’s review,^9^ approximately invasive fungal infections stand at 6.5 million cases and 2.5 million attributable deaths annually. *C. albicans* is the most common fungus that causes human disease, and throughout its whole life cycle, this polymorphic fungus alternates between distinct single-called yeast and multicellular mycelial forms in mammalian hosts.^10^ It is a common commensal of the human microbiome that inhabits the female reproductive tract, the skin, the mouth, and the gastrointestinal tract of healthy individuals in the form of yeast.^11,12^ One of the largest obstacles to the successful prevention of viral illnesses is antibiotic resistance. Therefore, it is crucial to recognize the adaptive mechanisms that bacteria utilize for withstanding minimal antibiotic stress to comprehend the processes that result in antibiotic resistance. The Gram-positive bacterium *B. subtilis* exhibits ways to compensate for environmental stress and competition.^13^ Also, *Salinivibrio proteolytic* is a new curved-rod-shaped, slightly halophilic bacterial Gram-negative strain known as strain AF-2004T and is isolated from Bakhtegan Lake, a hypersaline lake in southern Iran that contains 17% (w/v) total salt.^14^ Furthermore, according to the latest report by the WHO on cancer, almost every family accounts for one in each death worldwide, making it a significant contributor to mortality. Around 20 million new cases and 9.7 million cancer-related deaths occurred worldwide in 2022 and will increase about 77% in 2050. Hence, it is important to develop new therapeutics to treat microbial pathogens and cancerous cells.

**Figure 1:**
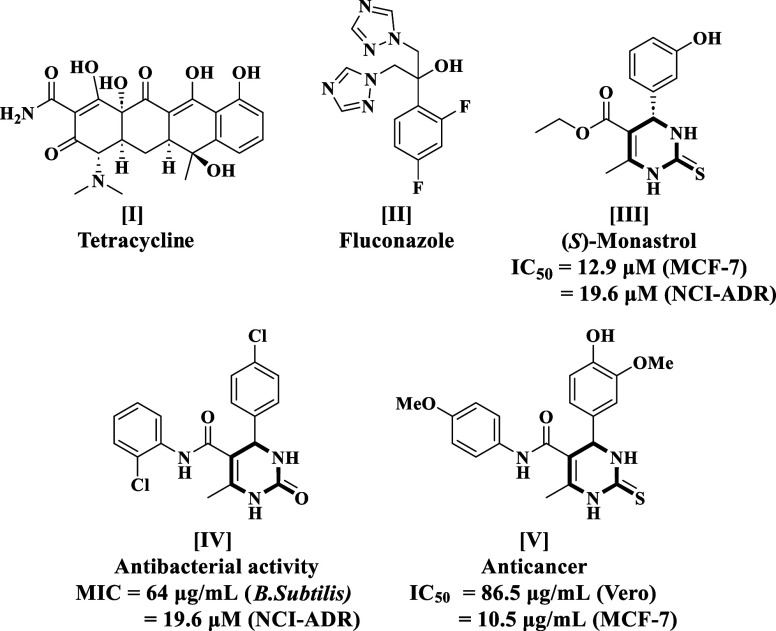
Some commercially available medications and physiologically significant 2-thioxo/2-oxo-3,4-DHPMs with antibacterial and antitumor properties.

Nitrogen-bearing heterocycles are essential to human life and are broadly exist in nature due to their extensive potential medicinal application.^15^ Pyrimidine is widely distributed in nature and is a six-membered aromatic heterocycle bearing nitrogen atoms at the first and third positions, which is used by cells to form the building blocks of DNA and RNA as well as plays an important role in neurotransmission, nucleic acids, cell metabolism, as well as vascular smooth muscle regulation.^16^ Out of two basic isomers of pyrimidine (3,4-dihydropyrimidine-2(1*H*)-one and 5,6-dihydropyrimidine-2(1*H*)-one), only 3,4-DHPM has attracted substantial attention. 3,4-DHPMs were first synthesized by *P. Benelli* via a one-pot three-component reaction of aryl aldehyde, urea, and β-ketoester in ethanol in the presence of an acid catalyst.^17^ 3,4-DHPM-5-carboxanilide scaffolds are a unique chemical motif that appeals to chemical developments in both synthetic and therapeutic contexts owing to their broad spectrum of pharmacological aspects such as antimicrobial, anticancer, antimycobacterial, anti-HIV, and anti-inflammatory effects. Some antibiotic-marketed drugs bearing the 2-thioxo/2-oxo-3,4-DHPM scaffold such as broad-spectrum polyketide antibiotic **I**,^18^ triazole-based broad-spectrum antifungal agent and antiproliferative **II**,^19^ (*S*)-Monastrol **III**,^20^**IV**,^21^ and **V**(22) are shown in Figure 1. Thereafter, the TDHPM-5-carboxanilides were synthesized by utilizing diverse reaction conditions^22−26^ by changing reagents and catalysts and reaction temperature. But these methodologies were suffered from various drawbacks, i.e., corrosive and potentially hazardous catalysts, harsh reaction conditions, longer reaction time, and required column chromatography to get the pure product. In our recently published article,^31^ a novel analogue of (*R*/*S*)-2-thioxo-THPM-5-carboxanilides has been synthesized through one-pot treatment of diverse aryl aldehydes with thiourea and various acetoacetanilide derivatives using taurine as a green bioorganic catalyst in ethanol at 100 °C. Thereafter, all the newly synthesized compounds were characterized using different spectroscopic techniques and were separated into an individual (*R*)- and (*S*)-isomer by using preparative liquid chromatography (prep-LC).

In continuation of our research toward the development of biologically active heterocyclic compounds,^27−30^ in this study, we aim to investigate the 3D field-based QSAR, antimicrobial, antiproliferative, molecular docking, and pharmacophore modeling of previously synthesized and enantiomerically pure (*R*/*S*)-TDHPM-5-carboxanilides.^31^ The goal is to investigate their potential as antibacterial and antifungal agents and to explore their antiproliferative properties. By using advanced computational techniques, we aim to identify the pharmacophoric features and binding affinities of these compounds and predict their physicochemical, pharmacokinetic, and ADMET profiles. Furthermore, this study will also investigate the antiproliferative properties of the compounds against solid tumor cell lines and explore their potential mechanism of action through live cell imaging and kinetics study. This work provides insights into the therapeutic potential of these compounds and their suitability for further development.

## 2 Results and Discussion

### 2.1 Chemistry

In continuation with our previously published research,^31^ in which the synthesized novel analogues of (*R*/*S*)-TDHPM-5-carboxanilides **4**(**a–w**) were synthesized through a one-pot, three-component reaction of different aromatic aldehydes **1**(**a**–**d**) with thiourea **2** and diverse acetoacetanilides **3**(**a**–**g**) using taurine as a green bioorganic solvent in ethanol at 100 °C. The newly synthesized **4**(**a**-**w**) were then isolated as pure (*R*)- and (*S*)-isomers using prep-LC, as well as their absolute configurations were confirmed through single-crystal X-ray diffraction and circular dichroism (CD) analysis. The substrate scope for the synthesis of **4**(**a–w**) as well as the percentage purity of each separated isomers is described in Table 1. All newly synthesized compounds were obtained with isolated yields of up to 99%. Furthermore, the isolated pure isomers achieved purities of up to 99.99%.

**Table 1:**
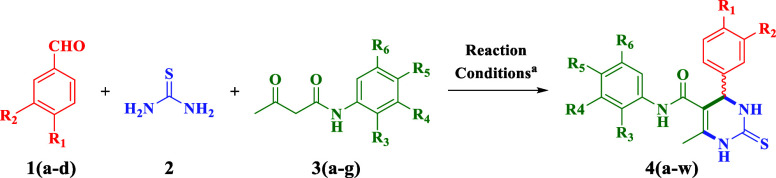
Substrate Scope for TDHPM-5-carboxanilides^a^^,^^b^^,^^c^

				% purity^c^	
entry	R_1_, R_2_	R_3_, R_4_, R_5_, R_6_	yield^b^ (*R*/*S*) (%)	(*R*)-isomer [**4**(**a–w**)]	(*S*)-isomer [**4**(**a′–w′**)]
4a	R_1_ = –OH; R_2_ = –H	R_3_ = –Cl; R_4_ = R_5_ = R_6_ = –H	92	97.98	99.63
4b	R_1_ = –OH; R_2_ = –OMe	R_3_ = –Cl; R_4_ = R_5_ = R_6_ = –H	93	98.04	99.52
4c	R_1_ = –OMe; R_2_ = –OMe	R_3_ = –Cl; R_4_ = R_5_ = R_6_ = –H	91	99.81	99.96
4d	R_1_ = –OH; R_2_ = –OH	R_3_ = –Cl; R_4_ = R_5_ = R_6_ = –H	99	99.58	99.78
4e	R_1_ = –OH; R_2_ = –H	R_4_ = –Cl; R_3_ = R_5_ = R_6_ = –H	96	95.20	99.72
4f	R_1_ = –OH; R_2_ = –OMe	R_4_ = –Cl; R_3_ = R_5_ = R_6_ = –H	97	91.92	99.17
4g	R_1_ = –OMe; R_2_ = –OMe	R_4_ = –Cl; R_3_ = R_5_ = R_6_ = –H	97	99.67	99.94
4h	R_1_ = –OH; R_2_ = –H	R_3_ = –Me; R_4_ = R_5_ = R_6_ = –H	94	96.59	99.70
4i	R_1_ = –OH; R_2_ = –OMe	R_3_ = –Me; R_4_ = R_5_ = R_6_ = –H	93	96.83	98.62
4j	R_1_ = –OMe; R_2_ = –OMe	R_3_ = –Me; R_4_ = R_5_ = R_6_ = –H	94	98.76	98.85
4k	R_1_ = –OH; R_2_ = –OH	R_3_ = –Me; R_4_ = R_5_ = R_6_ = –H	93	98.44	93.85
4L	R_1_ = –OH; R_2_ = –H	R_5_ = –Me; R_3_ = R_4_ = R_6_ = –H	87	99.17	99.92
4m	R_1_ = –OH; R_2_ = –OMe	R_5_ = –Me; R_3_ = R_4_ = R_6_ = –H	90	98.30	99.90
4n	R_1_ = -OMe; R_2_ = -OMe	R_5_ = –Me; R_3_ = R_4_ = R_6_ = –H	81	98.18	99.83
4o	R_1_ = –OH; R_2_ = –OH	R_5_ = –Me; R_3_ = R_4_ = R_6_ = –H	91	99.69	99.99
4p	R_1_ = –OH; R_2_ = –H	R_3_ = R_5_ = –OMe; R_4_ = R_6_ = –H	96	99.10	99.99
4q	R_1_ = –OMe; R_2_ = –OMe	R_3_ = R_5_ = –OMe; R_4_ = R_6_ = –H	81	99.80	99.95
4r	R_1_ = –OH; R_2_ = –OH	R_3_ = R_5_ = –OMe; R_4_ = R_6_ = –H	98	99.47	99.90
4s	R_1_ = –OH; R_2_ = –H	R_3_ = R_6_ = –OMe; R_5_ = –Cl; R_4_ = –H	96	99.71	99.82
4t	R_1_ = –OMe; R_2_ = –OMe	R_3_ = R_6_ = –OMe; R_5_ = –Cl; R_4_ = –H	98	99.30	99.57
4u	R_1_ = –OH; R_2_ = –H	R_4_ = –CF_3_; R_3_ = R_5_ = R_6_ = –H	93	95.51	93.72
4v	R_1_ = –OH; R_2_ = –OMe	R_4_ = –CF_3_; R_3_ = R_5_ = R_6_ = –H	95	80.33	94.17
4w	R_1_ = –OMe; R_2_ = –OMe	R_4_ = –CF_3_; R_3_ = R_5_ = R_6_ = –H	92	93.49	93.37

a Reaction conditions: aryl aldehydes **1**(**a–d**) (1.0 mmol), thiourea **2** (1.5 mmol), and acetoacetanilides **3**(**a–g**) (1.0 mmol), 15 mol % taurine in 4 mL of EtOH.

b Isolated yield.

c Purity (%).

### 2.2 Biological Application

#### 2.2.1 Antimicrobial Activity

In the present work, the antimicrobial and antifungal activities of previously synthesized and isolated (*R*)- and (*S*)-2-thioxo-3,4-dihydropyrimidine (TDHPM)-5-carboxanilides samples were studied against two different bacterial and fungal strains. Antimicrobial activity was determined using the agar well diffusion method^32,33^ and assessed based on the inhibition zone at different concentrations of samples **4**(**a–w**)-(*R*) as well as **4**(**a**′–**w**′)-(*S*)-isomers. Each of the isolated compounds showed that the zone of inhibition was composed with the activity of positive control tetracycline (30 μg/mL) for bacteria and fluconazole (25 μg/mL) for fungus. In all samples, significant growth inhibition occurred on bacterial strains and fungal stains. Tables 2 and 3 show the inhibition results of 100 μg/mL of **4**(**a–w**)-(*R*) as well as **4**(**a**′–**w**′)-(*S*)-isomers with a higher zone of inhibition after the incubation period.^34,35^ Here, the separated isomer with the highest zone of inhibition is the most potent.

**Table 2:**
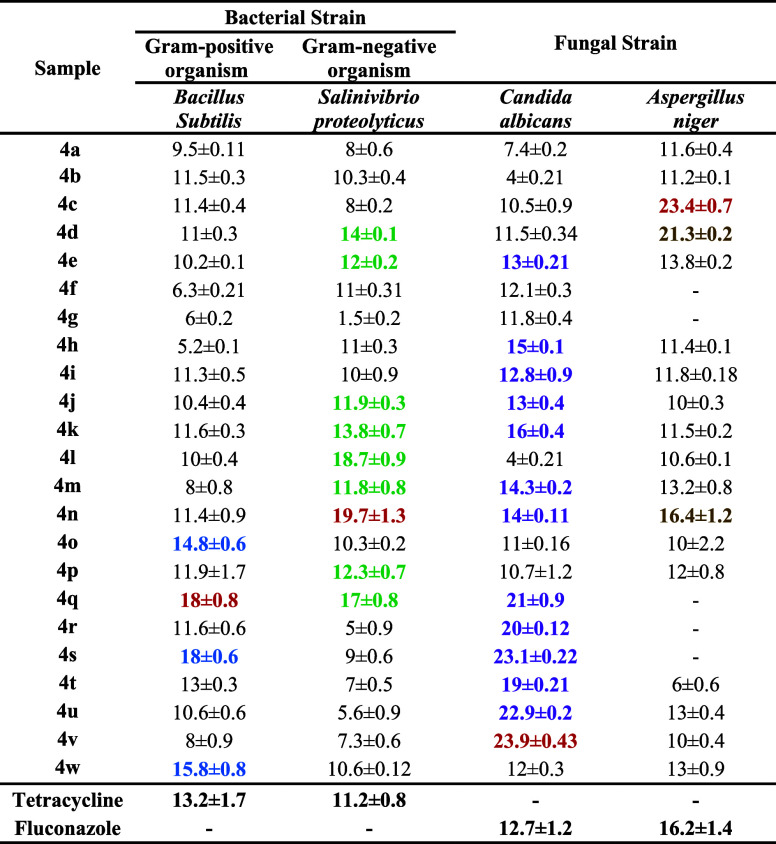
Antimicrobial Activity of **4**(**a–w**)’s Pure (*R*)-Isomer^a^

a Note: **Bold** and **dark red** values show more potential than tetracycline and fluconazole against *B. subtilis*, *S. proteolyticus*, *C. albicans*, and *A. niger*; **bold** and **blue** values show more potential than tetracycline against *B. subtilis*; **bold** and **dark green** values show more potential than tetracycline against *S. proteolyticus*; **bold and purple** values show more potential than fluconazole against *C. albicans*; **bold** and **gold accent 4** values show more potential than fluconazole against *A. niger*; and — not determined. Abbreviation: *B. subtilis,Bacillus subtilis*; *S. proteolyticus,Salinivibrio proteolyticus*; *C. albicans,Candida albicans*; and *A. niger,Aspergillus niger.*

**Table 3:**
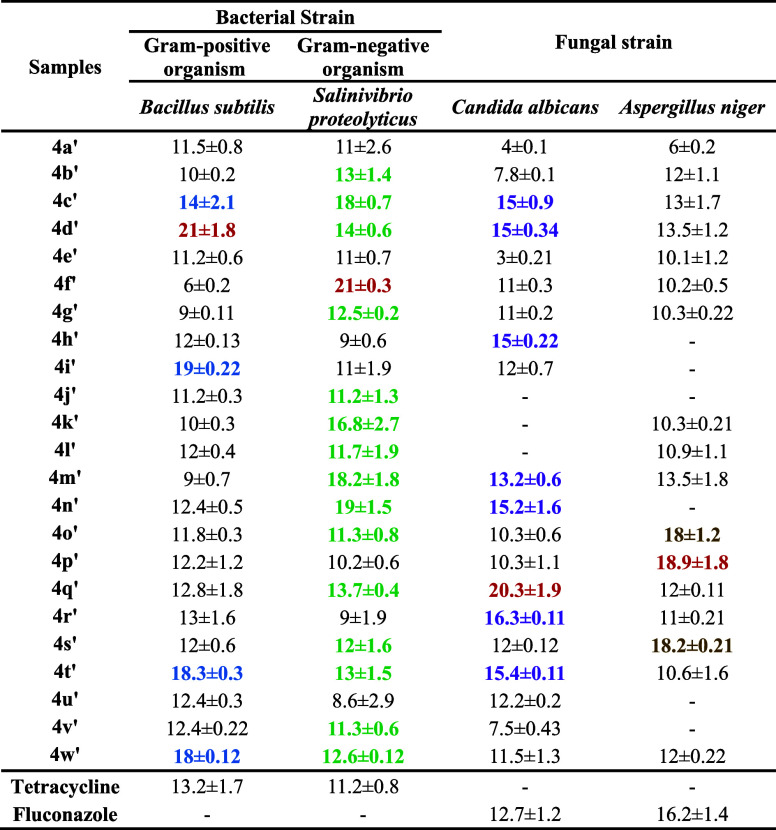
Antimicrobial Activity of 4(a′–w′)’s Pure (*S*)-Isomer^a^

a Note: **Bold** and **dark red** values show more potential than tetracycline and fluconazole against *B. subtilis*, *S. proteolyticus*, *C. albicans*, and *A. niger*; **bold** and **blue** values show more potential than tetracycline against *B. subtilis*; **bold** and **dark green** values show more potential than tetracycline against *S. proteolyticus*; **bold and purple** values show more potential than fluconazole against *C. albicans*; **bold** and **gold accent 4** values show more potential than fluconazole against *A. niger*; and —not determined. Abbreviation: *B. subtilis*, *Bacillus subtilis*; *S. proteolyticus*, *Salinivibrio proteolyticus*; *C. albicans*, *Candida albicans*; and *A. niger*, *Aspergillus niger.*

It can be observed from Tables 2 and 3 that compounds **4q**, **4n**, **4v**, and **4c** of pure (*R*)-isomer and **4d**′, **4f**′, **4q**′, and **4p**′ of pure (*S*)-isomer show the highest inhibition zone against Gram-positive and Gram-negative bacterial and fungal strains, respectively. Compounds **4q**-(*R*)- **and 4d**′-(*S*)-isomers show the highest zone of inhibition than the **4**(**o**, **s**, and **t**)-(*R*)-isomer and 4(c′, i′, t′, and w′)-(*S*)-isomers against Gram-positive organism *B. Subtilis*. The compounds **4n**-(*R*)- and **4f**′-(*S*)-isomers were found to be more potent than **4**(**d**, **e**, **k**, **l**, **m**, **p**, and **q**)-(*R*)-isomers and **4**(**b**′, **c**′, **d**′, **g**′, **j**′, **k**′, **l**′, **m**′, **n**′, **o**′, **q**′, **s**′, **t**′, **v**′, and **w**′)-(*S*)-isomers, respectively, against Gram-negative organism *Salinivibrio proteolyticus*. Compounds **4**(**b**, **e**, **h**, **i**, **j**, **k**, **m**, **n**, **q**, **r**, **s**, **t**, **u**, and **v**)-(*R*)-isomer, as well as **4**(**c**, **d**, **h**, **m**, **n**, **q**, **r**, and **t**)-(*S*)-isomer, respectively, were found to be more potent with the higher zone of inhibition than fluconazole against the fungal strain *C. Albicans*. Among them, **4v and 4q**′ were the most potent than others. Compounds **4**(**c**, **d**, **n**), as well as **4**(**o**′, **p**′, **s**′) with the higher zone of inhibition, were found to be more potent than fluconazole against the fungal strain *A. Niger* from which **4c and 4p**′ were found to be the most potent than the others. In the case of bacterial strains, *B. Subtilis* and *S. proteolyticus*, the (*S*)-isomers were found to be comparatively more potent than the (*R*)-isomer. While in the case of fungal strains, *C. Albicans* and *A. Niger*, (*R*)-isomers were found to be significantly more potent than the (*S*)-isomers.

#### 2.2.2 Antiproliferative Study

**Figure 2:**
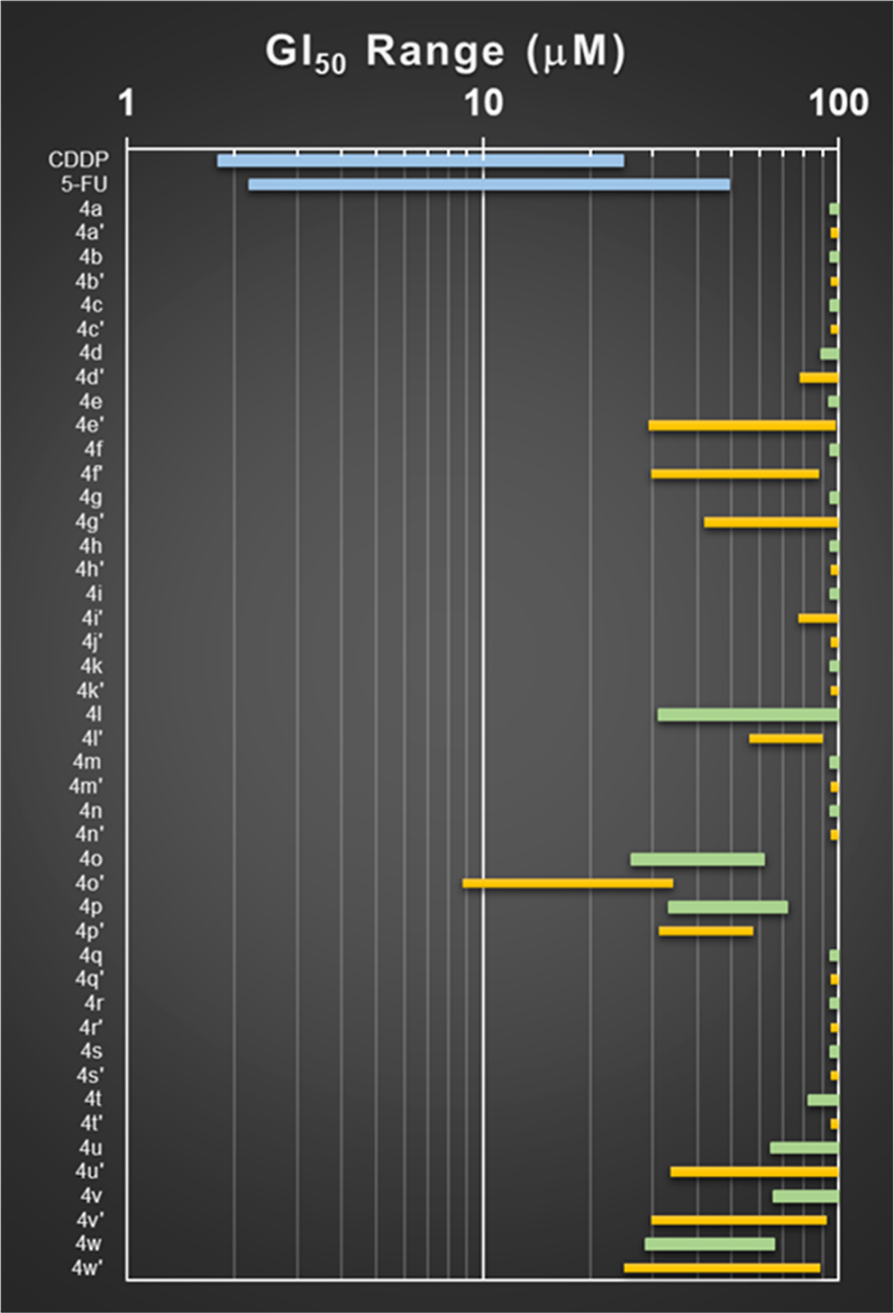
Range plot of GI_50_ values for compounds **4**(**a–w**). Blue bars: standard anticancer drug cisplatin (CDDP) and 5-FU. Green bars: *R* series. Yellow bars: *S* series.

Human tumor cell lines represent a fast and convenient tool to screen libraries of compounds in anticancer drug discovery campaigns. In this work, we used our standard panel of six human solid tumor cell lines representing relevant types of cancer. The antiproliferative effects (50% growth inhibition, GI_50_) of compounds **4**(**a–w**) against the cell lines were determined using the NCI protocol.^36^Figure 2 shows the results. Overall, the majority of the compounds were inactive. When comparing the enantiomeric series, the *S* compounds displayed a better activity profile. The most active compound of the series **4o′** was less potent that the standard anticancer drugs cisplatin and 5-fluorouracil (5-FU) (used as positive controls).

Table 4 lists the GI_50_ values obtained for those compounds that showed activity in at least one cell line. The lead compound **4o**′ induced antiproliferative effects in all cell lines in the range 8.8–34 μM.

**Table 4:** Antiproliferative Activity (GI_50_, μM) against Human Solid Tumor Cell Lines^a^

	cell lines (origin)					
samples	*A*549	*HeLa*	*MIA* PaCa-2	*SW*1573	T-47D	*WiDr*
**4d**(*R*)	90 ± 15	>100	>100	>100	>100	>100
**4d′**(*S*)	78 ± 31	>100	>100	>100	>100	>100
**4e**(*R*)	94 ± 8.0	>100	>100	>100	>100	>100
**4e′**(*S*)	29 ± 11	47 ± 14	68 ± 1.4	78 ± 14	98 ± 2.5	58 ± 22
**4f′**(*S*)	79 ± 36	66 ± 30	84 ± 27	88 ± 31	82 ± 31	30 ± 12
**4g′**(*S*)	42 ± 16	87 ± 19	>100	>100	>100	>100
**4i′**(*S*)	77 ± 0.5	>100	>100	>100	>100	>100
**4L**(*R*)	>100	97 ± 4.9	84 ± 19	59 ± 11	>100	31 ± 5.6
**4L′**(*S*)	74 ± 27	59 ± 24	91 ± 9.1	59 ± 8.2	71 ± 30	56 ± 18
**4o**(*R*)	36 ± 17	26 ± 9.7	42 ± 0.8	44 ± 10	61 ± 9.1	36 ± 11
**4o′**(*S*)	**21** ± **9**.**7**	**8**.**8** ± **3**.**1**	**34** ± **10**	**18** ± **4**.**5**	**26** ± **8**.**1**	**12** ± **5**.**1**
**4p**(*R*)	43 ± 14	33 ± 13	56 ± 18	72 ± 32	38 ± 11	51 ± 11
**4p′**(*S*)	38 ± 14	31 ± 4.5	58 ± 17	40 ± 5.7	53 ± 21	54 ± 8.5
**4t**(*R*)	>100	88 ± 14	>100	82 ± 23	>100	>100
**4u**(*R*)	85 ± 26	65 ± 32	>100	>100	88 ± 10	82 ± 26
**4u′**(*S*)	99 ± 1.0	34 ± 10	83 ± 15	>100	84 ± 27	78 ± 36
**4v**(*R*)	89 ± 18	65 ± 31	>100	>100	>100	91 ± 15
**4v′**(*S*)	34 ± 2.4	32 ± 2.7	34 ± 0.4	92 ± 11	36 ± 7.3	30 ± 0.8
**4w**(*R*)	30 ± 11	29 ± 11	45 ± 15	52 ± 11	66 ± 32	52 ± 22
**4w′**(*S*)	25 ± 7.3	45 ± 20	77 ± 13	89 ± 9.9	73 ± 24	44 ± 16

a Values represent mean ± standard deviation of two to three independent experiments.

**Figure 3:**
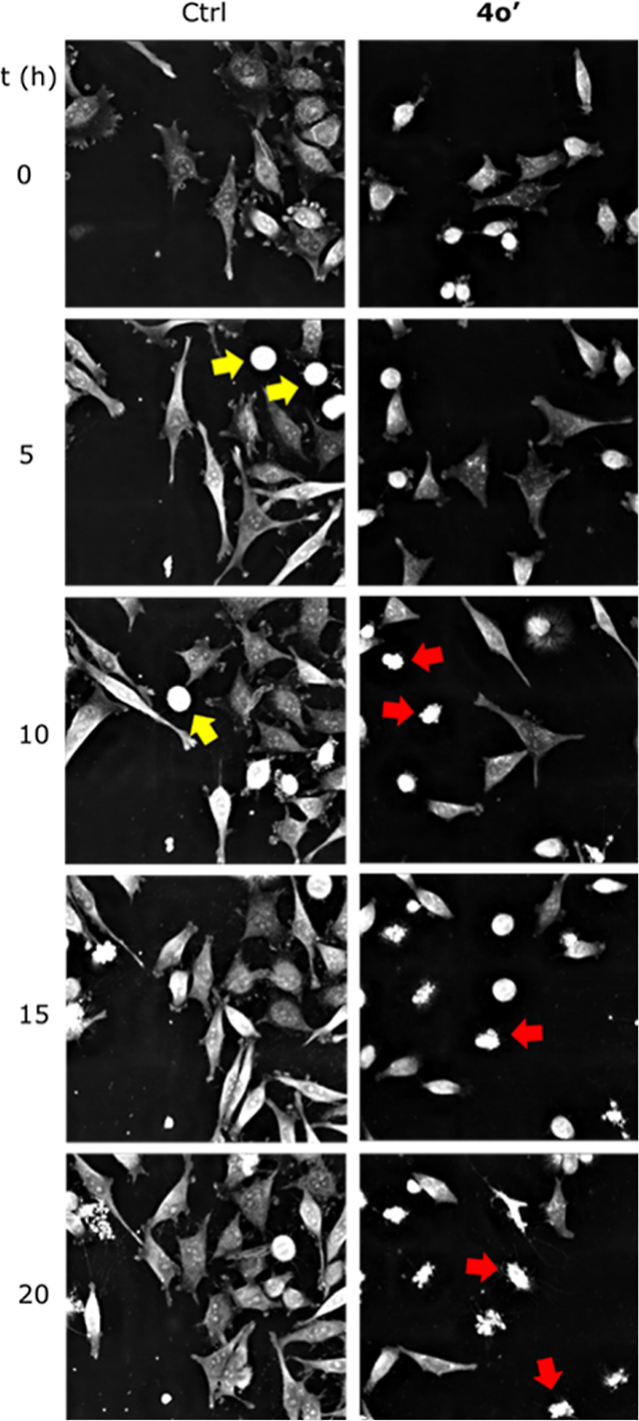
Representative snapshots of *HeLa* cells exposed to compounds **4o**′ (20 μM, 20 h). Yellow arrows: cells undergoing mitosis. Red arrows: apoptotic cells.

**Figure 4:**
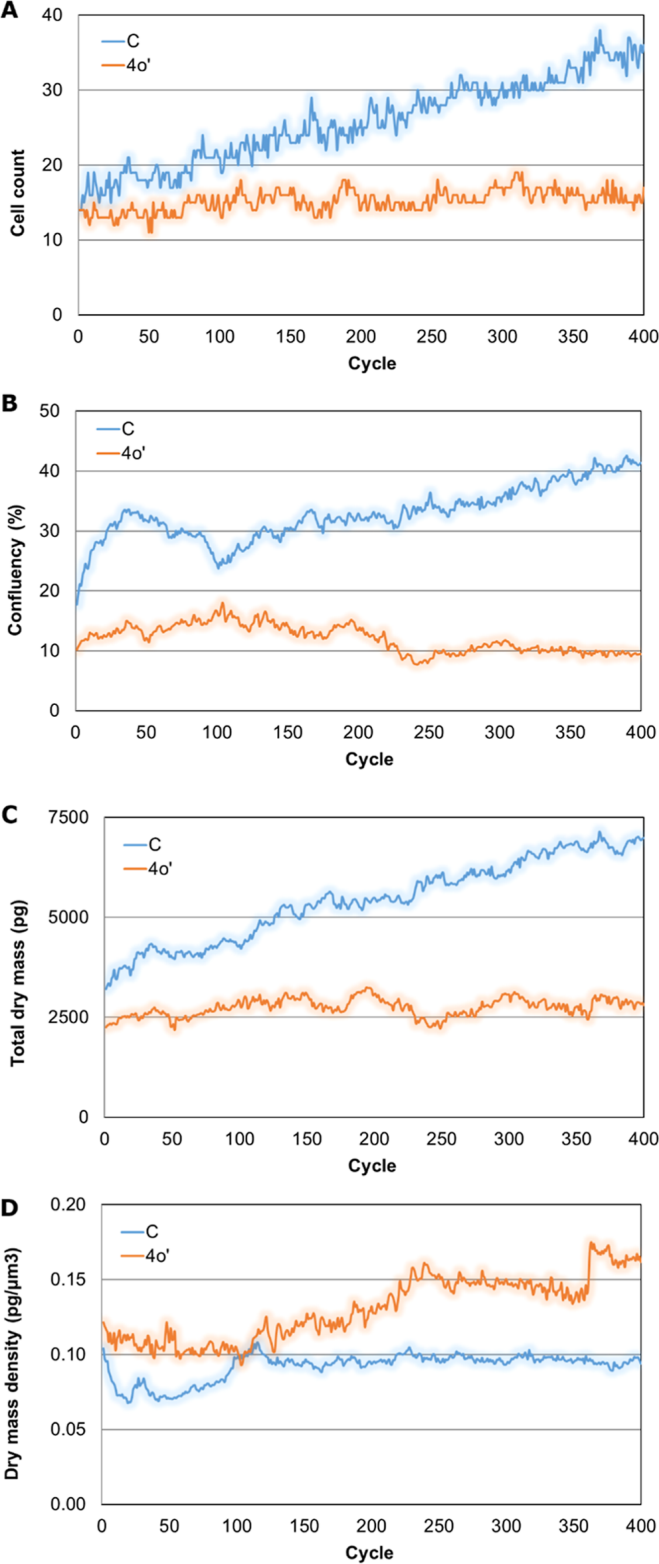
Kinetics of cell count (A), confluency (B), TDM (C), and DMD (D) of *HeLa* cells untreated or treated with **4o**′ (20 μM, 20 h).

Next, we examined the effects of **4o′** on *HeLa* cells by continuous live cell imaging (Supporting Information files Videos S1 and S2). With this technique, it is possible to follow over time the phenotypic changes induced in the cells by the compounds. Figure 3 shows snapshots of *HeLa* cells exposed for 20 h to a dose of 20 μM of **4o**′ (monitored every 3 min). The compound was able to induce apoptosis after 10 h of exposure. This cell event is characterized by a series of hallmarks, which induce cell shrinkage, nuclear condensation, and subsequent fragmentation (Figure 3, red arrows). The segmentation analysis provided kinetic phenotypic information supporting these observations (Figure 4). Cell counts (Figure 4A) and confluency (Figure 4B) show that the population of cells grew during the experiment. However, the population of treated cells did not increase. These results indicate that compound **4o′** prevents cell division from the start of the treatment. Total dry mass (TDM) is proportional to the number of existing cells, and an increase in TDM is a sign of cell proliferation. The kinetics of TDM (Figure 4C) show that compound **4o′** stopped the growth of the cell population, as denoted by a constant TDM. Apoptosis induces the collapse of cells, which produces cell shrinkage, and consequently and subsequently, dry mass density (DMD) increases. Changes over time on DMD (Figure 4D) show that cells treated with compound **4o′** started to die after 10 h of exposure.

### 2.3 Molecular Docking

Molecular docking is considered an important aspect of drug discovery, improving the identification and optimization of potential drug candidates.^37−39^ These studies provide predictive insights into ligand–target interactions for the discovery of novel compounds by supporting structure-based drug design. The capability of docking to enable high-throughput virtual screening of compound libraries accelerates hit identification. It also aids in understanding pharmacology, which is crucial in the fight against complex diseases. Recent developments also enable consideration of both ligand and receptor flexibility, thus enhancing the prediction accuracy. Besides, molecular docking is less expensive and diminishes the number of laboratory tests needed, which shortens the whole process of drug development.^40−44^ H-bonding, polar, and hydrophobic interactions play significant roles in molecular docking simulations. H-bonds can be very important in the stabilization of protein–ligand complexes and significantly contribute to the binding affinity. Hydrophobic interactions, occurring between nonpolar regions of molecules, drive the docking process and have been estimated in many cases to account for a large proportion of the contact area between proteins and ligands. Polar interactions, including electrostatic attractions, further enhance the stability and specificity of molecular recognition. A subtle balance of these interactions is required for the correct prediction of binding modes and affinities in docking simulations. Optimization of hydrophobic interactions and hydrogen bonding at the target–ligand interface often yield improved drug design results. Correct understanding and modeling of these interactions is important for the development of efficient computational tools useful in drug discovery and protein–ligand studies.

**Figure 5:**
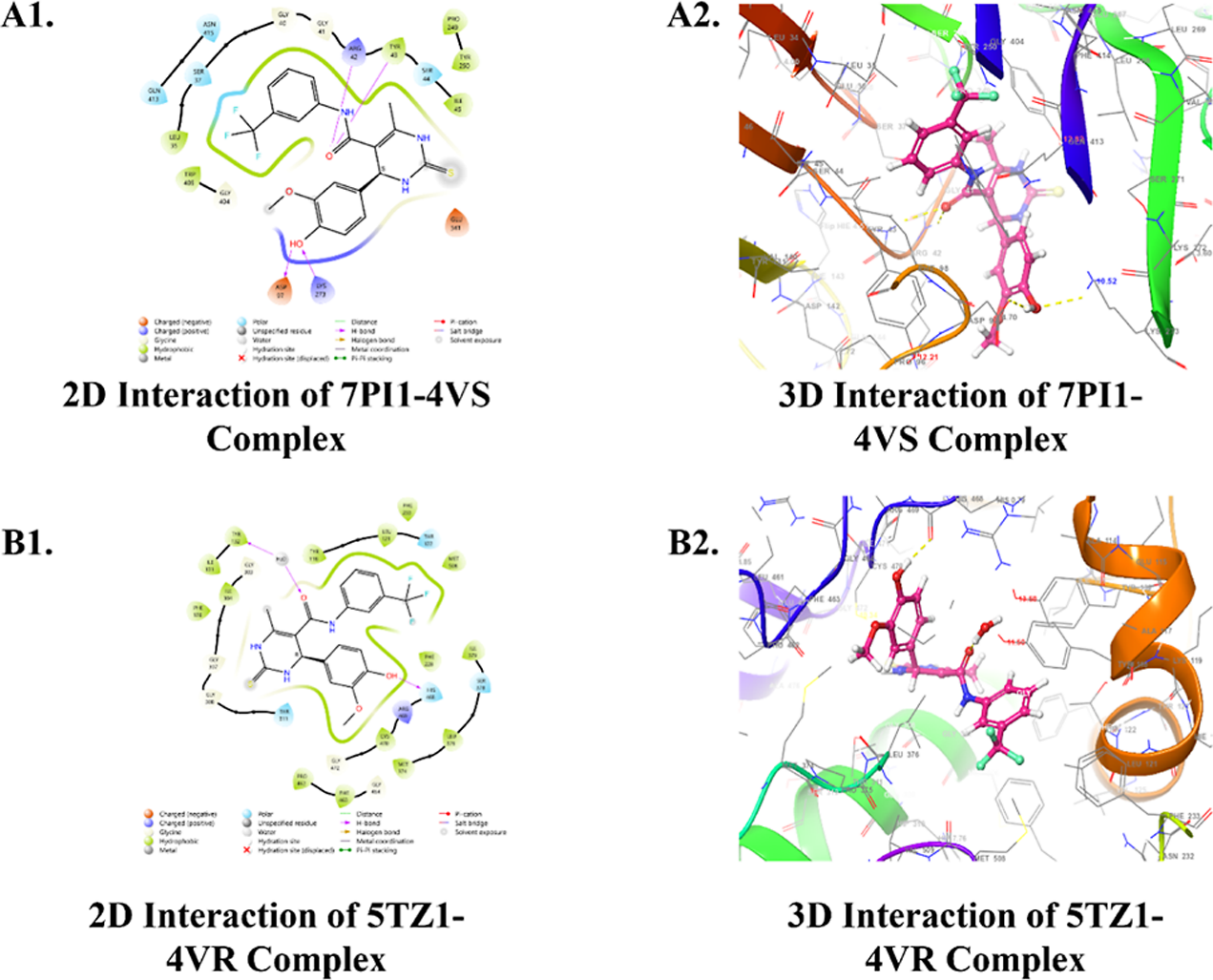
Ligand interaction 2D and 3D diagrams of molecular docking of 7PI1-**4v**-(*S*)-isomer (A1, A2) and 5TZ1-**4v**-(*R*)-isomer (B1, B2).

The results of molecular docking revealed that all the synthesized compounds possessed a good binding affinity toward *B. subtilis* PabB, PDB ID: 7PI1,^45^ with Glidescore from −8.607 to −1.857 kcal/mol. In the case of the docking against an antifungal target (*C. albicans* sterol 14-alpha demethylase PDB ID: 5TZ1),^46,47^ the compounds possessed the Glidescores between −10.261 and −6.234 kcal/mol. Also, the Glidescores of compounds were found to be comparable to those of the standard drugs (Supporting Information Table S1). The docking against PDB ID: 7PI1, the Glidescore of the compound that showed the best antibacterial property [**4v**-(*S*)-isomer] was found to be −8.607 kcal/mol, and it formed four H-bonds, with the residues Arg42, Tyr43, Asp97, and Lys273 of this target, respectively. The compound docked well inside the hydrophobic binding pocket created by amino acid residues Leu35, Tyr43, Ile45, Pro249, Tyr250, and Trp405. It also formed polar interaction with Ser37, Ser44, Gln413, and Asn415 residues (Figure 5A1,A2).

On the other hand, the Glidescore of the compound that showed the best antifungal property [**4v**-(*R*)-isomer] against PDB ID: 5TZ1 was found to be −10.261 kcal/mol, and it formed three H-bonds with Tyr132 and His468 residues. It was found to dock well within the hydrophobic pocket consisting of Tyr118, Leu121, Phe126, Ile131, Tyr132, Phe228, Phe233, Ile304, Met374, Leu376, Ile379, Pro462, Phe463, Cys470, and Met508 residues (Figure 5B1,B2). It also formed polar interactions better than the standard drug fluconazole (Table 4). The interacting residues of the experimentally determined best antibacterial [**4v**-(*S*)-isomer] and antifungal [**4v**-(*R*)-isomer] have been mentioned in Table 5 and Figure 5A1,A2,B1,B2.

**Table 5:** Interacting Residues of the Top Binding Energy-Containing Antibacterial [**4v**-(*S*)-Isomer] and Antifungal Leads [**4v**-(*R*)-Isomer] against Their Respective Targets

compounds	PDB ID	H-bond interactions	polar interactions	hydrophobic interactions
**4v**-(*S*)-isomer	7PI1	Arg42, Tyr 43, Asp97, Lys273	Ser37, Ser44, Gln413, Asn415	Leu35, Tyr43, Ile45, Pro249, Tyr250, Trp405
**4v**-(*R*)-isomer	5TZ1	Tyr132, His468	Thr122, Thr311, Ser378, His468	Tyr118, Leu121, Phe126, Ile131, Tyr132, Phe228, Phe233, Ile304, Met374, Leu376, Ile379, Pro462, Phe463, Cys470, Met508

### 2.4 Binding Free Energy Calculation and Induced-Fit Docking

The binding free energies^48−50^ of the protein–ligand complexes formed by the important compounds with the protein targets were estimated by utilizing the MM-GBSA panel of Schrödinger 2023_1. The results of MM-GBSA calculations are tabulated in Table 6.

**Table 6:** Binding Free Energies of Top Binding Energy-Containing Antibacterial [**4v**-(*S*)-Isomer] and Antifungal Leads [**4v**-(*R*)-Isomer] against Their Respective Targets

compounds	target PDB	MMGBSA-dG Bind (kcal/mol)
**4v**-(*S*)-isomer	7PI1	–22.68
**4v**-(*R*)-isomer	5TZ1	–47.06

**Figure 6:**
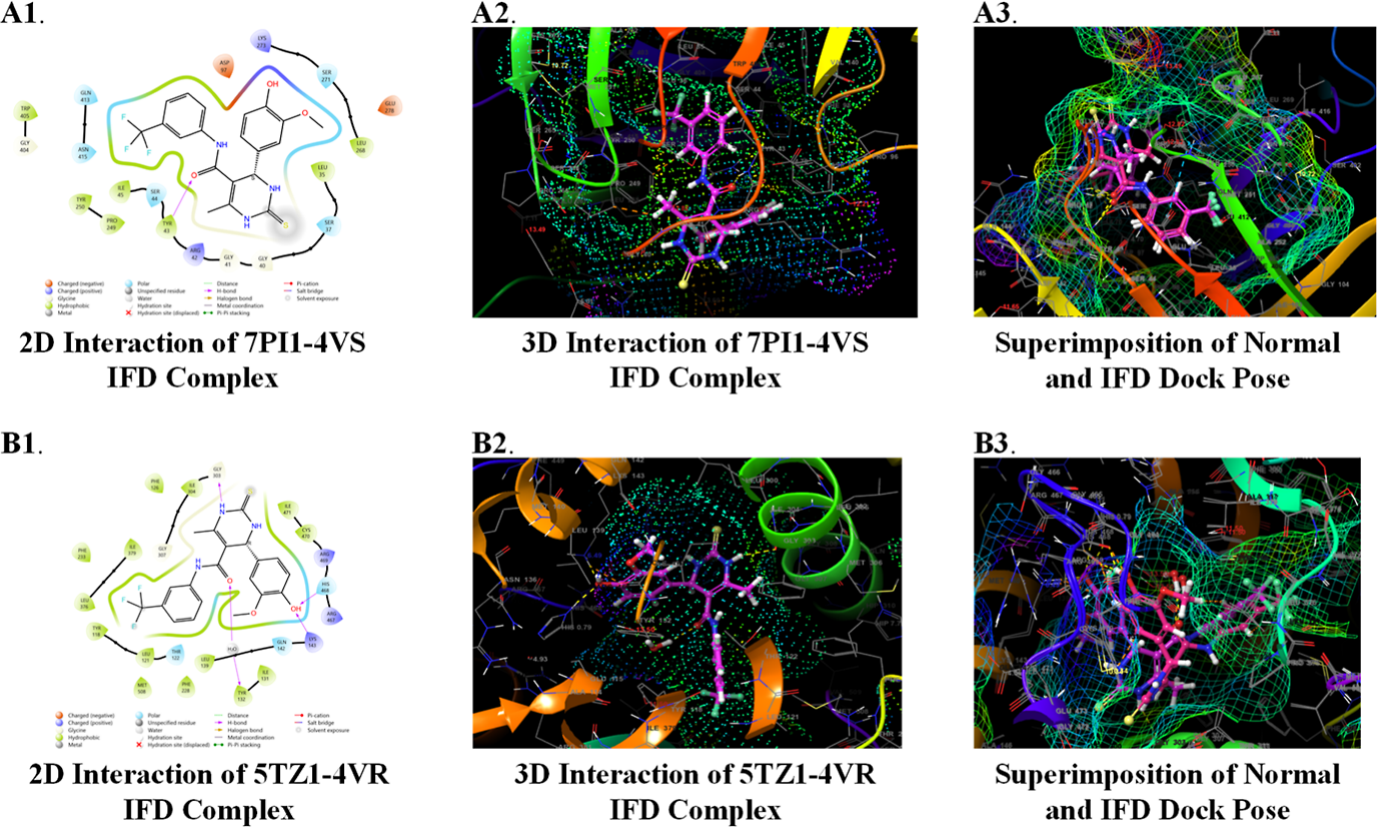
Ligand interaction 2D and 3D diagrams and superimposition of induced-fit docked pose (pink) with the standard pose (violet) for 7PI1-**4v**-(*S*)-isomer (A1, A2, A3) and 5TZ1-**4v**-(*R*)-isomer (B1, B2, B3).

To check the overall fitting and stability of the target–ligand complexes, the induced-fit docked 3D poses and the usual XP docking predicted 3D poses were overlapped. It was found that both of the poses were found to perfectly overlap into the binding pocket of 7PI1 (Figure 6A1,A2,A3), whereas in the case of 5TZ1’s binding pocket, both the docked poses were found to not be superimposed with each other (Figure 6B1,B2,B3). The results of induced-fit docking are tabulated in Table 7. For the 7PI1-**4v**-(*S*)-isomer complex (bacterial target), the Glidescore was improved from −8.607 to −9.179 kcal/mol, and the IFDScore was found to be −1008.40. The number of H-bonds had been decreased from four to one, and the amino acid residue was found to be Tyr43. On the other hand, the number of polar interactions had been increased from four to five, and the additional residue was Ser271. Moreover, the additional residue was found to be Leu268 (Figure 6A).

**Table 7:** Results of Induced-Fit Docking

compounds	Glidescores against PDB ID: 7PI1 (kcal/mol)	IFDScore	Glidescores against PDB ID: 5TZ1 (kcal/mol)	IFDScore
**4v**-(*S*)-isomer	–9.179	–1008.40		
**4v**-(*R*)-isomer			–11.272	–1032.49

On the other hand, in the case of the 5TZ1-**4v**-(*R*)-isomer complex (fungal target), the Glidescore had been improved from −10.261 to −11.272 kcal/mol, and the IFDScore was found to be −1032.49. The number of H-bond interactions had been increased from three to five in the induced-fit-docking-generated pose, and Lys143 and Gly303 were found to be the additional residues. However, there is a small number of polar interacting residues in the IFD-generated pose. Additionally, the number of hydrophobic residues was also decreased from 15 to 14, where Leu139 and Ile471 were the newly found hydrophobic residues in the IFD-generated pose (Figure 6B).

### 2.5 Pharmacophore Modeling

Pharmacophores may be described as the arrangement of a molecule that stores the essential characteristics necessary for the biological or pharmacological interaction of drugs. It is used for the virtual screening of molecules that trigger a biological response.^51^ A pharmacophore model usually contains two procedures, ligand-based and structure- or receptor-based.^51^ In the following two subsections, ligand-based and the protein–ligand complex-based (structure-based) pharmacophore modeling studies of the best docking score-containing antibacterial [7PI1-4v-(*S*)] and antifungal 5TZ1-4v-(*R*) compounds were taken into account.

#### 2.5.1 Using Ligand

**Figure 7:**
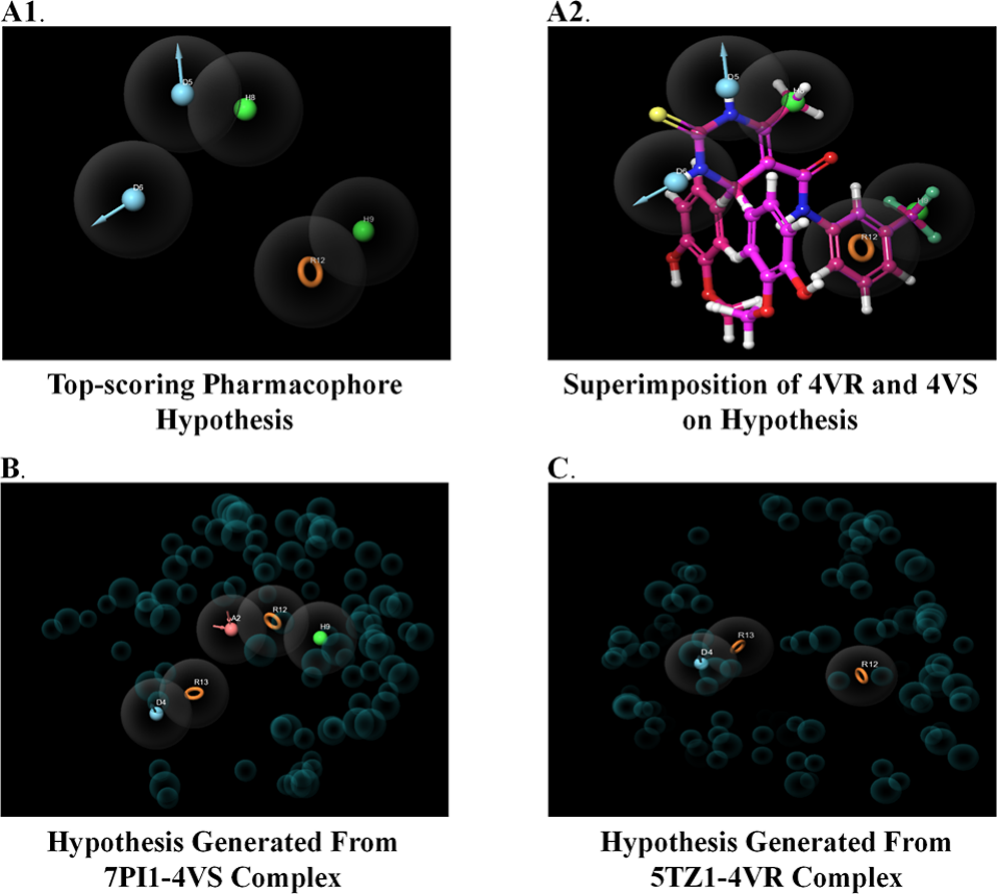
Top scoring pharmacophore hypothesis (A1), superimposition of **4v**-(*S*)- and **4v**-(*R*)-isomer on the hypothesis (A2), hypothesis generated from the 7PI1-**4v**-(*S*)-complex (B), and hypothesis generated from the 5TZ1-**4v**-(*R*)-complex (C).

In ligand-based pharmacophore modeling, a set of active molecules is superposed in order to capture the chemical activities that are needed for biological activities. Ligand-based pharmacophore modeling provides a number of advantages: the ability to develop models in the absence of the target protein structure, enabling the virtual screening of large compound libraries based on common features from known active ligands. This allows for the identification of fundamental chemical features that are important for biological activity and aids in lead optimization. It is also limited by the quality and diversity of the input data, which could reflect itself in the model accuracy. The performance on unknown bioactive conformations of ligands is difficult, and insights at the molecular level are less deep when compared to structure-based approaches.^52^ The results of pharmacophore modeling revealed that the best pharmacophoric hypothesis was found to be composed of two donors, two hydrophobic, and one aromatic feature (DDHHR) (Figure 7A1,A2). A total of 20 hypotheses were created, whose comprehensive data have been tabulated in Table 8. Several scoring functions describe the quality and the prediction of the hypotheses from a Phase pharmacophore model. These are Survival Score: A description for the robustness of a pharmacophore model. A high value will indicate a better survival model. Ideal survival scores of well-validated models will go usually above 2.5. Site Score refers to the quality of alignment of the pharmacophore features with active compounds, normally ranging between 0 and 1. The higher the site score, the better the alignment. Vector Score refers to the geometric arrangement of the pharmacophoric features; higher scores mean a more favored configuration for binding. Volume Score calculates the spatial volume occupied by the pharmacophoric features; this helps in assessing the extent to which a compound can fit into a binding site. The score range for both the vector and volume score is −1.0 to 1.0. From these ranges, it is visible that all of the generated were in the acceptable range. Boltzmann enhanced discrimination of receiver operating characteristic (BEDROC) score assesses the enrichment of active compounds in virtual screening results. Higher values are indicative of better performance in distinguishing between active and inactive compounds. The PhaseHypoScore score epitomizes the overall quality of the pharmacophore model, condensing several performance metrics into a single-value representative of its predictive capability. These scores jointly support the evaluation and improvement of pharmacophore models in an effort toward effective drug discovery and design.^53^

**Table 8:** Phase-Generated All Pharmacophoric Hypotheses

hypothesis	survival score	site score	vector score	volume score	BEDROC score	PhaseHypoScore
DDHHR_1	4.984	0.999942	1.000	0.575	1.000	1.299
DDHHR_2	4.978	0.999954	1.000	0.569	1.000	1.299
DDHHR_3	4.974	0.999964	1.000	0.575	1.000	1.298
DDHHR_4	4.971	0.999943	1.000	0.575	1.000	1.298
DDHHR_5	4.971	0.999962	1.000	0.575	1.000	1.298
DDHHR_6	4.965	0.999958	1.000	0.569	1.000	1.298
DDHHR_7	4.965	0.999960	1.000	0.566	1.000	1.298
DDHHR_8	4.961	0.999961	1.000	0.566	1.000	1.298
DDHHR_9	4.946	0.999962	1.000	0.575	1.000	1.297
DHHHR_1	4.917	0.999947	1.000	0.575	1.000	1.295
DDHR_1	4.424	0.999964	1.000	0.575	1.000	1.265
DDHR_2	4.423	0.999937	1.000	0.575	1.000	1.265
DDHR_3	4.417	0.999960	1.000	0.569	1.000	1.265
DDHR_4	4.414	0.999967	1.000	0.566	1.000	1.265
DHHR_1	4.407	0.999945	1.000	0.575	1.000	1.264
DHHR_2	4.403	0.999958	1.000	0.575	1.000	1.264
DHHR_3	4.400	0.999960	1.000	0.569	1.000	1.264
DHHR_4	4.399	0.999968	1.000	0.575	1.000	1.264
DHHR_5	4.397	0.999952	1.000	0.575	1.000	1.264
DHHR_6	4.395	0.999980	1.000	0.575	1.000	1.264

#### 2.5.2 Using Protein–Ligand Complex

On the other hand, in a structure-based method, a pharmacophore model is developed using the structural features of the protein and its complex with the ligand. The advanced *E*-pharmacophore^54^ model within Schrödinger uses energy-optimized structures from ligand–receptor complexes to identify crucial features that are important for biological activity, thus enabling the generation of pharmacophore hypotheses in the absence of active compounds. This model maps Glide XP energies onto the ligand atoms and thereby integrates the fragment docking results. Advantages include an improved predictive capability due to energy optimization, the possibility of its application in situations where active ligands are not available, and the capability for the discovery of new binding sites for drug design. On the other hand, it depends on high-quality structural data, generation of models may be complex, and capturing the dynamic nature of ligand–receptor interactions is difficult. Overall, despite the different advantages of an *E*-pharmacophore model in pharmacophore modeling, much attention has to be paid to data quality and computational resources. In this case, the results revealed that for the 7PI1-**4v**-(*S*)- complex, the *E*-pharmacophore was composed of two aromatic, one acceptor, one donor, and one hydrophobic (ADHRR) feature (Figure 7B). On the other hand, for the 5TZ1-**4v**-(*R*)-isomer complex, the *E*-pharmacophore was composed of two aromatic and one donor (DRR) feature (Figure 7C). These pharmacophoric features effectively contributed to the respective protein–ligand complex’s high binding affinity obtained from the molecular docking studies.

### 2.6 QSAR Study

#### 2.6.1 Results of AutoQSAR Analysis

**Figure 8:**
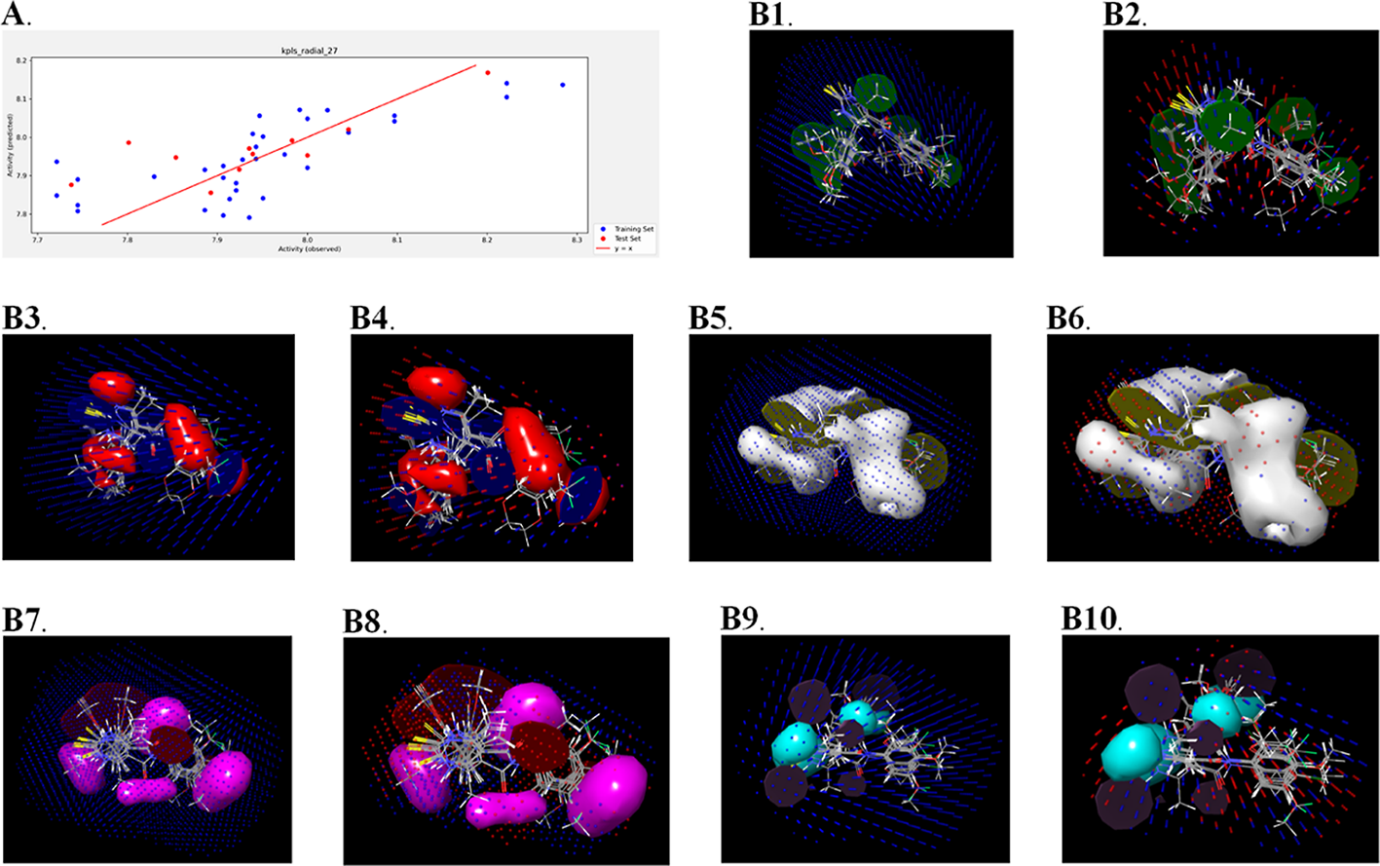
Results of QSAR analysis against bacterial strain. Scatter plot obtained from AutoQSAR (A); 3D field-based QSAR result visualization (B): contour map generated from Gaussian steric with the field intensity from Gaussian steric (B1), contour map generated from Gaussian steric with the field intensity from Gaussian electrostatic (B2), contour map generated from Gaussian electrostatic with the field intensity from Gaussian steric (B3), contour map generated from Gaussian electrostatic with the field intensity from Gaussian electrostatic (B4), contour map generated from Gaussian electrostatic with the field intensity from Gaussian steric (B5), contour map generated from Gaussian hydrophobic with the field intensity from Gaussian electrostatic (B6), contour map generated from the Gaussian H-bond acceptor with the field intensity from Gaussian steric (B7), contour map generated from Gaussian H-bond acceptor with the field intensity from Gaussian electrostatic (B8), contour map generated from the Gaussian H-bond acceptor with the field intensity from Gaussian steric (B9), and contour map generated from the Gaussian H-bond donor with the field intensity from Gaussian electrostatic (B10).

**Figure 9:**
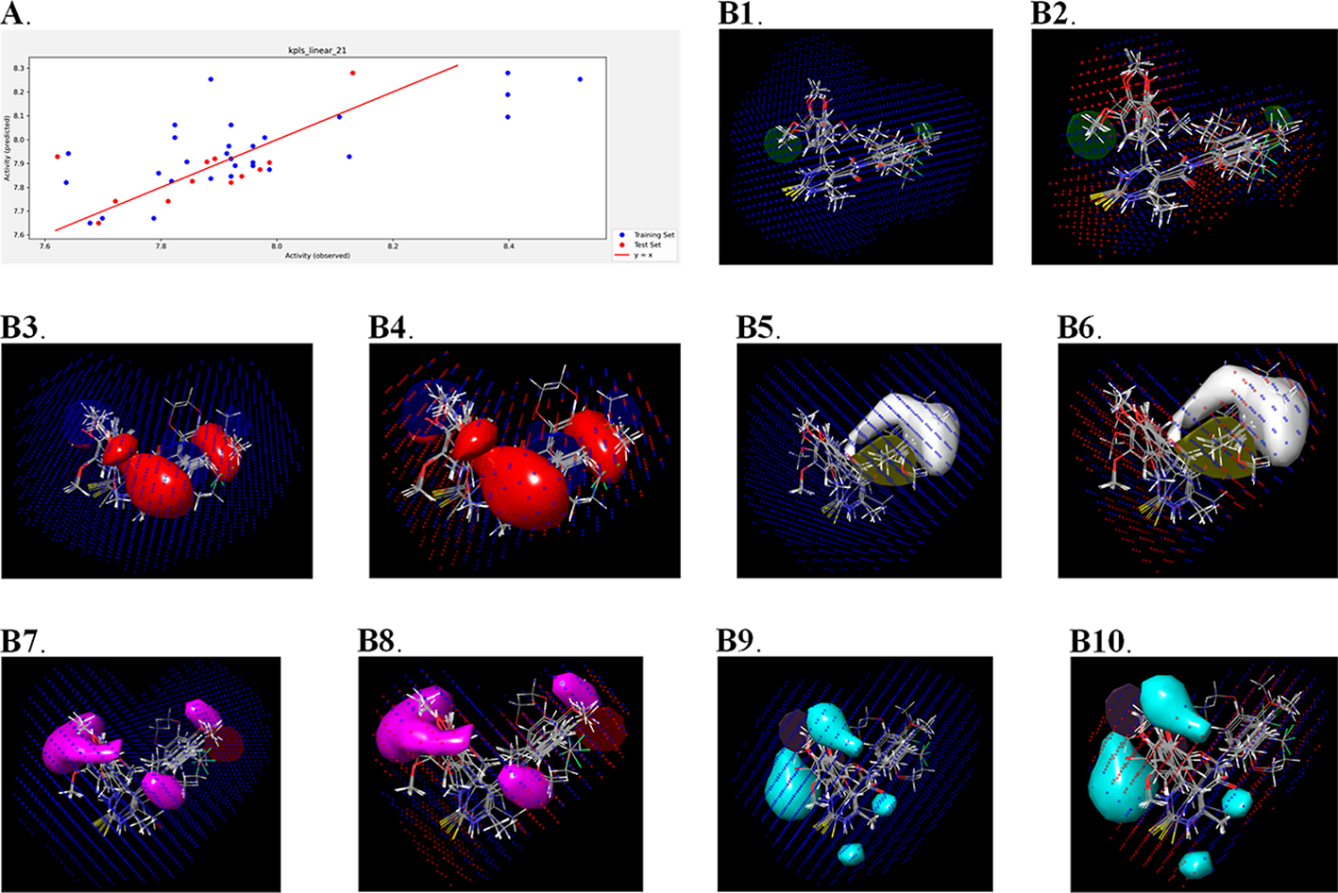
Results of QSAR analysis against fungal strain. Scatter plot obtained from AutoQSAR (A); 3D field-based QSAR result visualization (B): contour map generated from Gaussian steric with the field intensity from Gaussian steric (B1), contour map generated from Gaussian steric with the field intensity from Gaussian electrostatic (B2), contour map generated from Gaussian electrostatic with the field intensity from Gaussian steric (B3), contour map generated from Gaussian electrostatic with the field intensity from Gaussian electrostatic (B4), contour map generated from Gaussian hydrophobic with the field intensity from Gaussian steric (B5), contour map generated from Gaussian hydrophobic with the field intensity from Gaussian electrostatic (B6), contour map generated from the Gaussian H-bond acceptor with the field intensity from Gaussian steric (B7), contour map generated from the Gaussian H-bond acceptor with the field intensity from Gaussian electrostatic (B8), contour map generated from the Gaussian H-bond donor with the field intensity from Gaussian steric (B9), and contour map generated from the Gaussian H-bond donor with the field intensity from Gaussian electrostatic (B10).

The results of AutoQSAR analysis revealed that the top scoring model for the bacterial strain (*B. subtilis*) was a representative of a moderate model (having a *Q*^2^ > 0.5) and was found to be kpls (Kernel partial least-squares)_radial_27 with an overall score of 0.5970, a standard deviation (S.D.) of 0.0908, an *R*^2^ value of 0.5790, a root-mean-square error (RMSE) value of 0.0793, and a *Q*^2^ value of 0.5528. On the other hand, for the fungal strain (*C. albicans*), the top scoring model was found to be klps_linear_21 with an overall score of 0.5730, an SD value of 0.1597, an *R*^2^ value of 0.5226, an RMSE value of 0.1159, and a *Q*^2^ value of 0.2630. The scatter plots generated from these two models are depicted in Figures 8A and 9A.

#### 2.6.2 Results of 3D Field-Based QSAR

The results of both models [one for the bacterial strain (*B. subtilis*) and another for the fungal strain (*C. albicans*)] have been tabulated in the form of PLS in Tables 9 and 10. Additionally, the Gaussian field contributions in these models have also been tabulated in Supporting Information Tables S2 and S3, and the field contour maps have been depicted in Figures 8B1–8B10 and 9B1–9B10.

**Table 9:** PLS Statistics of the 3D Field-Based QSAR Model (against Bacterial Strain)^a^

# factors	1	2	3	4	5
SD	0.1250	0.1130	0.1016	0.0957	0.0915
*R*^2^	0.1558	0.3333	0.04809	0.5563	0.6099
*R*^2^CV	–0.0217	–0.1624	–0.2109	–0.2035	–0.1704
stability	0.942	0.713	0.537	0.466	0.465
*F*	5.4	7.0	8.3	8.2	7.8
*p*	0.028	0.00343	0.000438	0.000212	0.000152
RMSE	0.12	0.11	0.14	0.15	0.16
*Q*^2^	0.1152	0.2946	–0.2689	–0.4651	–0.6953
Pearson-r	0.3584	0.5466	0.2559	0.2625	0.1182

a Note: # factors, no. of PLS factors; SD, standard deviation; *R*^2^, correlation coefficient; *R*^2^CV, cross-validated value (leave-one-out validation); F, variance ratio; p, statistical significance; *Q*^2^, predictive relevance; and Pearson r, Pearson correlation coefficient.

**Table 10:** PLS Statistics of the 3D Field-Based QSAR Model (against Fungal Strain)^a^

# factors	1	2	3	4	5
SD	0.1860	0.1679	0.1448	0.1365	0.1322
*R*^2^	0.1999	0.3724	0.5511	0.6170	0.6555
*R*^2^CV	–0.1668	–0.0220	–0.0877	–0.1687	–0.1873
stability	0.745	0.787	0.582	0.509	0.493
*F*	6.7	7.7	10.2	9.7	8.8
*p*	0.015	0.00234	0.000141	8.36 × 10^–5^	9.2 × 10^–5^
RMSE	0.21	0.22	0.19	0.23	0.24
*Q*^2^	0.0860	–0.0168	0.2395	–0.1545	–0.2751
Pearson-r	0.3230	0.2291	0.5059	0.2481	0.2162

a Note: # factors, no. of PLS factors; SD, standard deviation; *R*^2^, correlation coefficient; *R*^2^CV, cross-validated value (leave-one-out validation); *F*, variance ratio; *p*, statistical significance; *Q*^2^, predictive relevance; and Pearson r, Pearson correlation coefficient.

### 2.7 Computational Physicochemical and Pharmacokinetic Property Prediction

**Figure 10:**
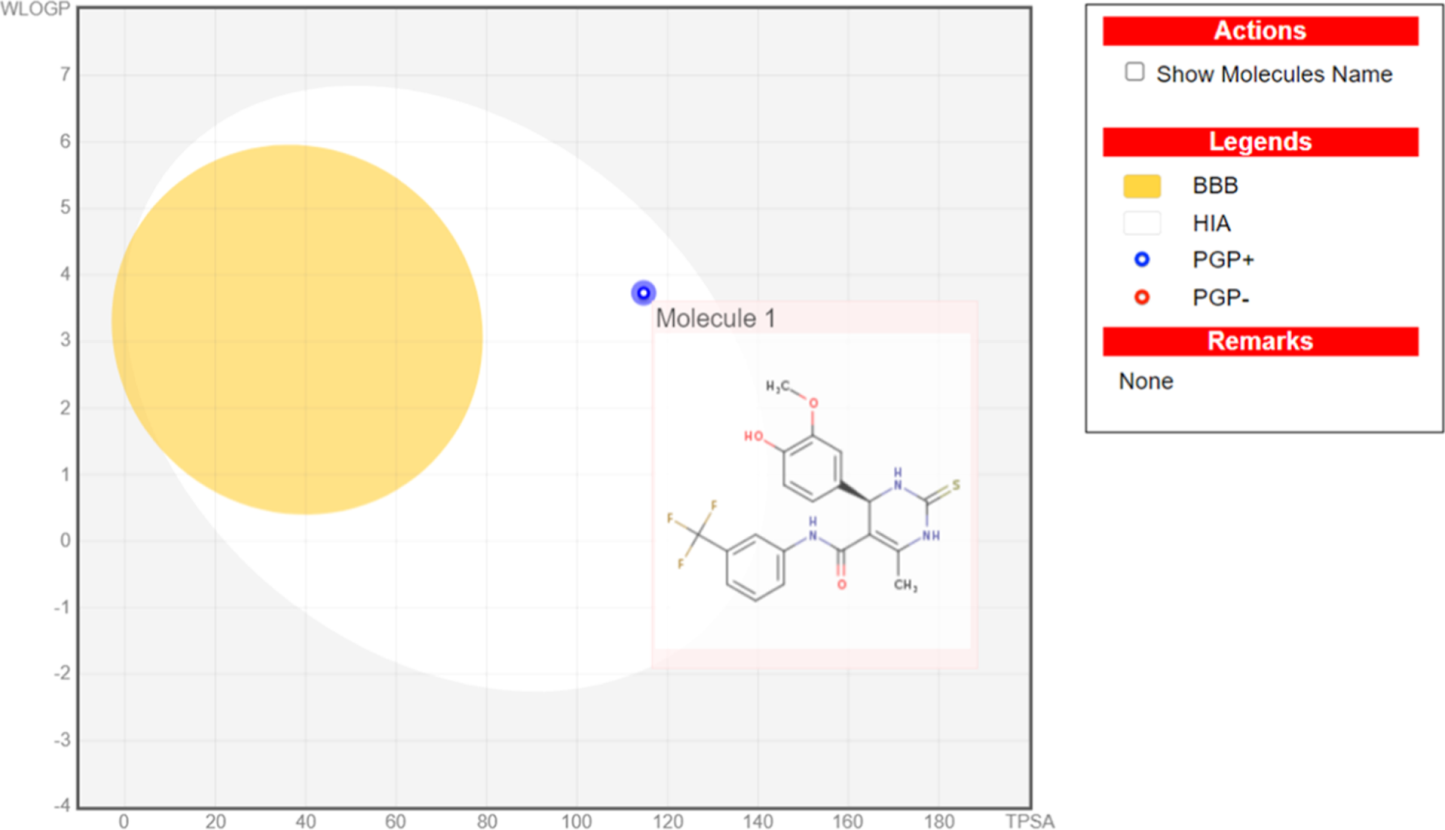
Egg-boiled diagrams of **4v**-(*S*)-isomer imported from SwissADME [compound **4v**-(*R*)-isomer was not visible in the egg-boiled diagram].

The results of the Qikprop program and SwissADME server have been summarized in Table 11. From the Qikprop-predicted data, the parameters summarized in Table 11 were found to be in the permissible range. On the other hand, the SwissADME online server provided an egg-boiled diagram (Figure 10), which revealed that the **4v**-(*R*)-isomer was predicted as non-CNS-active [the compound **4v**-(*S*)-isomer was not visible in the egg-boiled diagram, and hence, it was considered nonpredictable]. Both of them were predicted as a P-glycoprotein (PGP)-substrate. They exhibited no violation of Lipinski’s, Ghose’s, Veber’s, Egan’s, and Muegge’s (Bayer’s) rules. Out of these rules, Lipinski’s Rule of Five has generally been considered the most critical in drug discovery. It was developed by Christopher A. Lipinski in 1997 and provided guidelines for the likely oral bioavailability of new drug candidates with four entries: not more than five hydrogen bond donors, not more than ten hydrogen bond acceptors, a molecular mass below 500 Da, and a LogP not greater than five. General use of this rule in the prediction of oral bioavailability and pharmacokinetic properties of drug candidates and in this research depends a great deal on their success in clinical trials and market approval since it defines the important physicochemical properties that correlate with effective drug absorption and distribution within the human body. Both synthetic analogues were found to be the inhibitors of most of the cytochrome P450 enzymes (CYP), except for CYP1A2. Hence, they could be further tested for their pharmacokinetic and drug-likeness properties experimentally. All the derivatives show in-silico ADME profile below cutoff value which is quite suitable for the drug-likeness of these molecules.

**Table 11:** Predicted Physicochemical and Pharmacokinetic Properties of the Compounds **4v**-(*S*)- and **4v**-(*R*)-Isomers^a^

compounds	4v-(*S*)-isomer	4v-(*R*)-isomer
mol_MW	437.436	437.436
SASA	692.35	692.351
FOSA	170.28	170.281
FISA	109.11	109.11
PISA	214.521	214.521
volume	1232.746	1232.748
donor HB	2	2
accpt HB	4.5	4.5
glob	0.8030738	0.8030738
QP polrz	42.661	42.661
QP logPC16	12.243	12.243
QP logP oct	20.411	20.411
QP logP w	10.136	10.136
QP logP o/w	5.207	5.207
QP P Caco	914.562	914.562
QP log BB	–0.319	–0.319
QP P MDCK	5488.761	5488.761
QP log Kp	–2.391	–2.391
QP log Khsa	0.852	0.852
percent human oral absorption	100	100
PSA	93.746	93.746
Pgp substrate	yes	yes
CYP1A2 inhibitor	no	no
CYP2C19 inhibitor	yes	yes
CYP2C9 inhibitor	yes	yes
CYP2D6 inhibitor	yes	yes
CYP3A4 inhibitor	yes	yes

a **Note**: mol_MW, molecular weight (130/725); SASA, total SASA (300/1000); FOSA, hydrophobic SASA (0/750); FISA, hydrophilic SASA (7/330); PISA, carbon Pi SASA (0/450); donor HB, no. of donor hydrogen bonds (0/6); accpt HB, no. of acceptor hydrogen bonds (2/20); glob, globularity (0.75/0.95); QPpolrz, predicted polarizability (13/70), QP logPC16, predicted hexadecane/gas partition coefficient (4/18); QP logP oct, predicted octanol/gas partition coefficient (8/35); QP logP w, predicted water/gas partition coefficient (4/45); QP logP o/w, predicted octanol/water partition coefficient (−2/6.5); QP P Caco, Apparent Caco-2 Permeability (nm/s) (<25 poor, >500 great); QP log BB, predicted brain/blood barrier permeability (−3.0/1.2); QP P MDCK, Apparent MDCK Permeability (nm/s) (<25 poor, >500 great); QP log Kp, predicted skin permeability (Kp in cm/h); QP log K hsa, predicted serum protein binding (−1.5/1.5); PSA, vdW Polar SA (7/200); Pgp, P-glycoprotein; and Cyp, cytochrome P450.

## 3 Conclusions

In this study, we investigated 3D field-based QSAR, antimicrobial, antiproliferative, molecular docking, and pharmacophore modeling of previously synthesized enantiomerically pure (*R*/*S*)-TDHPM-5-carboxanilides. The best QSAR model was used to generate scatter plots for bacterial and fungal targets, *B. subtilis* and *C. albicans*, respectively. Pharmacophore modeling and molecular docking studies revealed that the pharmacophoric features of the best docking score-containing antibacterial [7pI1-4v’] and antifungal 5TZ1-4v compounds contributed to their high binding affinities of −8.607 kcal/mol (*B. subtilisPabB*; PDB: 7PI1) and −10.261 kcal/mol (*C. albicans* sterol 14α-demethylase; PDB: 5TZ1). These results were further supported by the induced field docking score of −1008.40 and −1032.49, and also both the poses were perfectly overlapped into each other in the binding cavity of 7PI1, as well as both isomers inhibited cytochrome P450. *In silico* ADMET profiles indicated favorable drug-likeness for these molecules, suggesting that compound **4v** may target *C. albicans* sterol 14-α demethylase. In vitro antimicrobial activity showed that enantiomerically pure isomers **4q**, **4d′**, **4n**, **4f′**, **4v**, **4q′**, **4c**, and **4p′** were more potent than tetracycline and fluconazole against *B. subtilis*, *S. proteolyticus*, *C. albicans*, and *Aspergillus niger*. Antiproliferative activity results showed that compound **4o′** exhibited GI50 values between 8.8 and 34 μM against six solid tumor cell lines. Following the greater potential of **4o′**, live cell imaging as well as kinetics of cell count, confluency, TDM, and DMD of *HeLa* cells untreated/treated with **4o′** was carried out. The goal of this work is to evaluate their potential as antibacterial and antifungal agents and to explore their antiproliferative properties. This work will provide insights into the therapeutic potential of these compounds and their suitability for further development.

## Data Availability

All the necessary files and tools to reproduce our work, including SwissADME, molecular docking, and QSAR protocols, are thoroughly described in the Supporting Information file.

## Supporting Information Available

General methods for the synthesis of (*R*/*S*)-2-thioxo-3,4-dihydropyrimidine(TDHPM)-5-carboxanilides; antimicrobial activity; antiproliferative activity; molecular docking; binding free energy calculation and induced-fit docking; pharmacological modeling; QSAR study; computational physicochemical and pharmacokinetic property prediction; and analytical and spectroscopic (^1^H NRM, ^13^C NMR, and HRMS) data of (*R*/*S*)-2-thioxo-3,4-dihydropyrimidine(TDHPM)-5-carboxanilides (PDF)
Control (AVI)
Treated with 4**o′** (AVI)

## Supplementary Material

ao4c09899_si_001.pdf

ao4c09899_si_002.avi

ao4c09899_si_003.avi
