# Supplementary material for: QSAR, Antimicrobial, and Antiproliferative Study of (R/S)-2-Thioxo-3,4-dihydropyrimidine-5-carboxanilides
Source: ACS Omega. 2025 Feb 10;10(7):7013–26. doi: 10.1021/acsomega.4c09899 (PMC11866182; doi:10.1021/acsomega.4c09899)
Supplement: Supplementary file 1 — ao4c09899_si_001.pdf [file ao4c09899_si_001.pdf]

Supporting Information

for

QSAR, Antimicrobial and Antiproliferative study of (*R*/*S*)-2-thioxo-3,4-dihydropyrimidine(TDHPM)-5-carboxanilides

Mehul P. Parmar[a], Anwesha Das[b], Disha P. Vala[a], Savan S. Bhalodiya[a], Chirag D. Patel[a], Shana Balachandran[c], Nagesh Kumar Kandukuri[d], Shreya Kashyap[e], Adam N. Khan[f], Aday González-Bakker[f], Madan Kumar Arumugam[c], José M. Padrón[f], Arijit Nandi[b,g], Sourav Banerjee[e,]*, Hitendra M. Patel[a,]**

[a] Department of Chemistry, Sardar Patel University, Vallabh Vidyanagar – 388120, Gujarat, India.
[b] Department of Pharmacy, Sanaka Educational Trust Group of Institutions (SETGOI), Department of Pharmacy, Malandighi, Durgapur, West Bengal 713212.
[c] Cancer biology lab, Center for Molecular and Nanomedical Sciences, Sathyabama Institute of Science and Technology, Chennai-600119, Tamil Nadu, India.
[d] YMC Application Lab, Plot no. 78/A/6, Phase VI, Industrial Park Jeedimetla, Gajularamaram Village, Quthbullapur, Medchal, Hyderabad - 500055, Telangana, India.
[e] Division of Cancer Research, School of Medicine, University of Dundee, Dundee, DD1 9SY, UK.
[f] BioLab, Instituto Universitario de Bio-Orgánica Antonio González, Universidad de La Laguna, Avda. Astrofísico Francisco Sánchez 2, 38206 La Laguna, Spain.
[g] Institute for Molecular Bioscience, The University of Queensland, 306 Carmody Road, St Lucia Qld 4072 Australia.

* Co-corresponding author: Sourav Banerjee, E-mail: s.y.banerjee@dundee.ac.uk

** Corresponding author: Hitendra M. Patel, E-mail: hm_patel@spuvvn.edu

Contents

1. General Methods 3

2. General Procedure for the synthesis of (*R*/*S*)-2-thioxo-3,4-dihydropyrimidine(TDHPM)-5-carboxanilides 3

3. Biological Screening 3

3.1 *In vitro* antimicrobial study 3

3.2 *In vitro* antiproliferative study 3

3.2.1 Human Cancer Cell Lines 3

3.2.2 Antiproliferative Assay 4

3.2.3 Label-Free Continuous Live Cell Imaging 4

3.3 Molecular Docking 4

3.4 Binding free energy calculation and induced-fit docking 4

3.5 Pharmacophore modelling 5

3.5.1 Using Ligands 5

3.5.2 Using Protein-ligand Complex 5

3.6 Quantitative Structure-Activity Relationship (QSAR) model development and validation .. 5

3.6.1 AutoQSAR 5

3.6.2 Three-dimensional (3D) field-based QSAR 5

3.7 Computational physicochemical and pharmacokinetic property prediction 6

References: 8

1. General Methods

All the reagents, buffer solutions and solvents with the corresponding quality used in synthesis, separation, antimicrobial and antiproliferative activity were purchased from TCI, Sigma-Aldrich, and Spectrochem Pvt. Ltd. and used without further purification.

2. General Procedure for the synthesis of (*R*/*S*)-2-thioxo-3,4-dihydropyrimidine(TDHPM)-5-carboxanilides

All the previously reported (*R*/*S*)-2-thioxo-3,4-dihydropyrimidine-5-carboxanilides were synthesized via a one-pot reaction of diverse aldehydes with thiourea and various acetoacetanilides and were isolated to their enantiomerically pure isomers using Prep-LC[1]. Also ^{1}H and ^{13}C NMR data as well as computational data of CD analysis were found from the SI file of our previous article[1] (DOI: https://doi.org/10.1039/D4RA01391C).

3. Biological Screening

3.1 *In vitro* antimicrobial study

In the antimicrobial assay, the prepared drugs were analysed for resistance against the Gram-positive organism (*Bacillus subtilis*) and the Gram-negative organisms (*Salinivibrio proteolyticus*) and the fungal strains *Candida albicans* and *Aspergillus niger*. The bacterial and fungal suspension was prepared in sterilized phosphate buffer solution (PBS) *p*H 7.0, and then the inoculum was adjusted to 10^7 spores/mL after counting in a cell counter chamber. Pure cultures of each bacterial and fungal strains were inoculated into brain heart infusion (BHI) broth and incubated for 4 h and from the BHI broth 100 µL of each strain were swabbed on individual Muller Hinton agar plates using sterile cotton swabs based on our previous report[2, 3]. Wells of 6 mm diameter were punched on Muller Hinton agar using gel puncture. Different concentrations of samples were dissolved in DMSO (**4**(**a**-**w**)-(*R*)-Isomer and **4**(**a'**-**w'**)-(*S*)-Isomer) as well as positive control, Tetracycline for bacterial strains, Fluconazole was used as a standard for antifungal and negative control (DMSO) controls were loaded on respective wells and then the plates were incubated for 3 days at 30 °C. After incubation, the inhibition zones were measured in mm to determine the efficiency of the samples[3, 4]. Moreover, different concentrations of **4**(**a**-**w**)-(*R*)-Isomer & **4**(**a'**-**w'**)-(*S*)-Isomer were evaluated as antibacterial and antifungal to detect the minimum inhibitory concentration (MIC).

3.2 *In vitro* antiproliferative study

3.2.1 Human Cancer Cell Lines

Cells used in this study were donated to the group by partner institutions. For screening, we used the following human solid tumour cell lines: A549 and SW1573 (non-small cell lung), MIA PaCa-2 (pancreas), T-47D (breast), HeLa (cervix), and WiDr (colon). Cells were grown in RPMI 1640 medium supplemented with 5% FBS and 2 mM L-glutamine. Cells were incubated in 60 mm Petri dishes at 37 °C, 5% CO2, and 95% relative humidity. The cell culture medium used was RPMI 1640 supplemented with 5% heat-inactivated FCS, 2 mM L-glutamine, 100 U/mL penicillin, and 0.1 mg/mL streptomycin. Cell cultures were passaged biweekly using 0.05% trypsin and maintained at low passage.

3.2.2 Antiproliferative Assay

DMSO was added to each sample to prepare 10 mM stock solutions. The tests were performed using our implementation of the NCI60 protocol[5] . Cells were grown in monolayers in 96-well plates. The maximum test concentration was 100 µM, and the sample exposure time was 48 h. The standard anticancer drugs cisplatin (CDDP) and 5-fluorouracil (5-FU) were used as positive controls.

3.2.3 Label-Free Continuous Live Cell Imaging

HeLa cells were seeded onto a 35 mm high glass-bottom µ-dish (IBIDI, Germany) at a density of 80,000 cells/dish. After 24 h, the growth medium was replaced with RPMI 1640 phenol red-free medium, and cells were treated with 20 µM of **4o'** for 20 h using the CX-A label-free cell imaging system (Nanolive S.A., Switzerland), and the status of cell populations was recorded every 10 min. The initial field of observation was selected considering a homogeneous distribution of cells (236 µm × 236 µm). After the acquisition, images were processed using Eve segmentation and analysis software (Nanolive S.A., Switzerland) to evaluate cell content and morphology parameters (Eve Analytics). The measurements were obtained for each population at each time point for every treatment.

Video S1_Control (1).avi

Video S1: Control

Video S2_Treated (1).avi

Video S2: Treated with **4o’**

3.3 Molecular Docking

For the purpose of the validation of the results of the in vitro antimicrobial activities, molecular docking studies of all the syntehsized compounds were done against one bacterial target (*Bacillus subtilis* PabB, PDB ID: 7PI1, 1.73 Å)[6, 7], and one fungal target (*Candida albicans* sterol 14-alpha demethylase [CYP51], PDB ID: 5TZ1, 2.00 Å)[8]. The targeted bacterial protein to *B. subtilis*, and the targeted fungal protein to *C. albicans* were imported from the PDB protein databank (https://www.rcsb.org/structure/7PI1, and https://www.rcsb.org/structure/5TZ1). At first, these proteins were retrieved in the “Protein Preparation Workflow”[9, 10] module of Schrödinger Maestro, and followed by, that the proteins were prepared by employing the three step procedure, namely, “Preprocess”ing the proteins (here, the missing loops were added), “Optimiz”ing the hydrogen bonds, and finally, “Minimiz”ing and “Delet”ing the water molecules. In these steps, the default parameters were utilized. Next, the ligands were prepared by employing the “LigPrep”[10] module of Schrödinger Maestro, where all the default parameters were applied for the preparation of the molecules. For molecular docking, the protocol of extra precision (XP) of Schrödinger 2023_1's Glide was applied, and in every case, the co-crystallized ligand was selected around which the grid parameters were defined[11-13].

3.4 Binding free energy calculation and induced-fit docking

To predict the binding free energy of the top antibacterial (**4v'**-(*S*)-isomer) and antifungal (**4v**-(*R*)-isomer) ligands (based on the binding energies), the Prime MM-GBSA module of Schrödinger was implemented. For the calculations, VSGB was taken as the solvation model, while the force field “OPLS4” (latest available) was taken for this calculation[14-17]

To validate the dynamics stability of the molecular docking complexes, induced-fit of the top antibacterial (**4v'**-(*S*)-isomer) and antifungal (**4v**-(*R*)-isomer) compounds was performed against the respective target proteins. For this purpose, Schrödinger's Induced-fit docking panel was applied, where the standard protocol and OPLS4 force field were utilized for induced-fit docking[18-21].

3.5 Pharmacophore modelling

3.5.1 Using Ligands

To generate the common pharmacophoric features of the top antibacterial (**4v'**-(*S*)-isomer) and antifungal (**4v**-(*R*)-isomer) compounds (based on the binding energies), the pharmacophore modelling was carried out. For this, the Phase module of Schrödinger Maestro was utilized, which is used to supply a couple of pharmacophoric features, that is, negative (N), positive (P), aromatic (R), hydrogen bond donor (D), acceptor (A), and hydrophobic (H). Here, multiple ligands were chosen to generate the pharmacophore hypothesis. Additionally, as the ligands were not previously aligned, hence, the option entitled "Find best alignment and common features" was chosen. The remaining parameters were kept as they were[22, 23].

3.5.2 Using Protein-ligand Complex

To perform the pharmacophore modelling using the "Protein-ligand Complex" option, 7PI1-**4v'**-(*S*) and 5HT1-**4v**-(*R*) complexes were selected. The "E-pharmacophore" or "Auto" method was selected, and other setting remained default[24].

3.6 Quantitative Structure-Activity Relationship (QSAR) model development and validation

3.6.1 AutoQSAR

AutoQSAR or automated QSAR panel is a machine learning (ML)-derived panel in Schrödinger software. Here, for building of the QSAR models, the "AutoQSAR" panel of Schrödinger release 2023-1 was employed. Schrödinger's AutoQSAR panel automates the creation, validation, and deployment of QSAR models with the help of machine learning. This tool was developed to make model generation, on a wide range of molecular properties, effortless for the most inexperienced novice user in the field of computational chemistry and drug discovery. Several important steps are automated within QSAR modeling, which involves the generation of descriptors, selection of features, and model generation. Thus, this automation enables the user to rapidly develop a series of models (usually 10) using various different machine learning methods and after that rank them according to their performance. For this, at first, the zone of inhibition values of all the synthesized compounds against the bacteria and the fungus (namely, *Bacillus subtilis*, and *Candida albicans*, respectively) were converted into p_zone of inhibition values (negative logarithms of the zone of inhibition). Followed by this, all the LigPrep-prepared synthesized molecules were aligned using the "Ligand Alignment" tool, and these ligands (datasets) were randomly assigned into training set (70%) and test set (30%). The top ten best scoring models were kept, and for the bacterial and fungal targets, out of these ten models, the best scoring models having the top statistical values were considered for generating the Scatter plot[25].

3.6.2 Three-dimensional (3D) field-based QSAR

Field-based QSAR is an *in-silico* method that establishes a correlation between the 3D structural features of a set of aligned molecules and their known biological activities. This approach is similar to the popular techniques such as Comparative Molecular Field Analysis (CoMFA) and Comparative Molecular Similarity Indices Analysis (CoMSIA), which also utilize the 3D structural information of compounds to build predictive QSAR models.

In this case, for building of the QSAR model, the "Field-Based QSAR" panel of Schrödinger release 2023-1 was employed, where all the LigPrep-prepared synthesized molecules were aligned using the "Ligand Alignment" tool, and these ligands (datasets) were randomly assigned into training set (70%) and test set (30%). Here, it is worth mentioning that the ligands were assigned based on the fact that both the training and test set should contain a diverse set of synthesized compounds. The "p_zone of inhibition" values were chosen as the activity property. For the generation of the model, a maximum of 5 partial least square (PLS) factors was considered, and the leave-one-out cross-validation method was employed for the validation of the QSAR model[26].

3.7 Computational physicochemical and pharmacokinetic property prediction

To predict the drug-likeness of the top antibacterial **4v**-(***S***)-, and antifungal **4v**-(***R***)-Isomer compounds (based on the binding energies), the in silico predicted pharmacokinetic and physicochemical properties were determined using the Qikprop module of Schrödinger Maestro (https://www.schrodinger.com/products/qikprop)[27], the online server entitled "SwissADME" (http://www.swissadme.ch/)[28, 29] were utilized.

Table 1. Docking score of the synthetic derivatives **4**(**a**-**w**)-(*R*)-isomers and **4**(**a'**-**w'**)-(*S*)-isomers against respective targets.

Compounds	Glidescores against PDB ID: 7PI1 (Kcal/mol)	Glidescore against PD ID: 5TZ1 (kcal/mol)
4a-(*R*)-	-5.051	-8.460
4b-(*R*)-	-5.159	-8.914
4c-(*R*)-	-5.472	-7.541
4d-(*R*)-	N/A	N/A
4e-(*R*)-	-4.943	-8.700
4f-(*R*)-	-5.995	-9.064
4g-(*R*)-	-5.153	-9.340
4h-(*R*)-	-5.583	-8.143
4i-(*R*)-	-5.358	-8.696
4j-(*R*)-	-4.819	-8.087
4k-(*R*)-	-6.437	-9.584
4l-(*R*)-	-4.901	-8.904
4m-(*R*)-	-5.352	-9.557
4n-(*R*)-	-4.885	-8.008
4o-(*R*)-	-6.718	-9.213
4p-(*R*)-	-5.965	-7.391
4q-(*R*)-	-4.460	-6.946
4r-(*R*)-	-4.973	-8.800
4s-(*R*)-	-5.641	-7.026
4t-(*R*)-	-5.805	-7.645
4u-(*R*)-	-5.666	-9.334
4v-(*R*)-	-6.217	-10.261

4w-(*R*)-	-7.048	-9.381
4a'-(*S*)-	-5.962	-9.841
4b'-(*S*)-	-6.688	-10.101
4c'-(*S*)-	-2.738	-9.706
4d'-(*S*)-	N/A	N/A
4e'-(*S*)-	-4.220	-9.296
4f'-(*S*)-	-4.107	-9.048
4g'-(*S*)-	-1.857	-9.115
4h'-(*S*)-	N/A	N/A
4i'-(*S*)-	-6.325	-10.001
4j'-(*S*)-	-6.010	-9.771
4k'-(*S*)-	-7.040	-9.693
4l'-(*S*)-	-4.419	-9.109
4m'-(*S*)-	-4.990	-9.273
4n'-(*S*)-	-2.913	-7.659
4o'-(*S*)-	-4.163	-10.013
4p'-(*S*)-	-3.440	-7.657
4q'-(*S*)-	-3.671	-6.690
4r'-(*S*)-	-5.292	-9.481
4s'-(*S*)-	-4.256	-8.519
4t'-(*S*)-	-3.671	-8.392
4u'-(*S*)-	-8.168	-6.234
4v'-(*S*)-	-8.607	-7.425
4w'-(*S*)-	-7.291	-9.021
Tetracycline	-5.605	-
Fluconazole	-	-5.514

N/A: Score not available

Table 2. Field contribution of Gaussian field (Against bacterial strain)

**#Factor**	**Gaussian steric**	**Gaussian electrostatic**	**Gaussian hydrophobic**	**Gaussian H-bond acceptor**	**Gaussian H-bond donor**
1	0.394214	0.089062	0.170247	0.204067	0.142410
2	0.435891	0.070321	0.186737	0.189966	0.117085
3	0.417921	0.070806	0.230372	0.172164	0.108736
4	0.431062	0.070026	0.230963	0.160838	0.107112
5	0.440913	0.073897	0.229507	0.142008	0.113676

Note: #Factors, No. of PLS factors

Table 3. Field contribution of Gaussian field (Against fungal strain)

#Factor	Gaussian steric	Gaussian electrostatic	Gaussian hydrophobic	Gaussian H-bond acceptor	Gaussian H-bond donor
1	0.411587	0.073443	0.222633	0.202884	0.089452
2	0.401094	0.066465	0.242429	0.217633	0.072379
3	0.368257	0.090267	0.290226	0.181481	0.069769
4	0.362253	0.096762	0.305194	0.156222	0.079570

5	0.350067	0.103481	0.312017	0.147107	0.087328

Note: #Factors, No. of PLS factors

References:

1. Parmar, M. P.; Vala, D. P.; Bhalodiya, S. S.; Upadhyay, D. B.; Patel, C. D.; Patel, S. G.; Gandholi, S. R.; Shaik, A. H.; Miller, A. D.; Nogales, J.; Banerjee, S.; Padrón, J. M.; Amri, N.; Kandukuri, N. K.; Patel, H. M., A green bio-organic catalyst (taurine) promoted one-pot synthesis of (*R*/*S*)-2-thioxo-3,4-dihydropyrimidine(TDHPM)-5-carboxanilides: chiral investigations using circular dichroism and validation by computational approaches. *RSC Adv.* **2024,** *14*(13), 9300-9313. https://doi.org/10.1039/d4ra01391c
2. Loo, Y. Y.; Rukayadi, Y.; Nor-Khaizura, M. A.; Kuan, C. H.; Chieng, B. W.; Nishibuchi, M.; Radu, S., In Vitro Antimicrobial Activity of Green Synthesized Silver Nanoparticles Against Selected Gram-negative Foodborne Pathogens. *Front. Microbiol.* **2018,** *9*, 1555. https://doi.org/10.3389/fmicb.2018.01555
3. Gnanasekaran, P.; Roy, A.; Sirpu Natesh, N.; Raman, V.; Ganapathy, P.; Arumugam, M. K., Removal of microbial pathogens and anticancer activity of synthesized nano-thymoquinone from Nigella sativa seeds. *Environ. Technol. Innovation* **2021,** *24*, 102068. https://doi.org/10.1016/j.eti.2021.102068
4. Geremew, A.; Carson, L.; Woldesenbet, S., Biosynthesis of silver nanoparticles using extract of Rumex nepalensis for bactericidal effect against food-borne pathogens and antioxidant activity. *Front. Mol. Biosci.* **2022,** *9*, 991669. 10.3389/fmolb.2022.991669
5. Adrián, P.; Alexis, R. G.; Roderick, A.; Kaylie, D.; Miguel, X. F.; Giovanna, B.; José, M. P., Naphthol-derived Betti bases as potential SLC6A14 blockers. *Int. J. Mol. Cell. Med.* **2019,** *2*(2), 35-40. https://doi.org/10.31083/j.jmcm.2019.02.7181
6. Rooms, L. D., Race, P.R., Devine, A., Willis, C.L., Back, C.R., Burton, N., Sudol, A., Crystal structure of Bacillus subtilis PabB, component 1.
7. Rooms, L. D., Race, P.R., Bacillus subtilis PabB. 7 September 2022 ed.; RCSB Protein Data Bank: 2021.
8. Hargrove, T. Y.; Friggeri, L.; Wawrzak, Z.; Qi, A.; Hoekstra, W. J.; Schotzinger, R. J.; York, J. D.; Guengerich, F. P.; Lepesheva, G. I., Structural analyses of *Candida albicans* sterol 14α-demethylase complexed with azole drugs address the molecular basis of azole-mediated inhibition of fungal sterol biosynthesis. *J. Biol. Chem.* **2017,** *292*(16), 6728-6743. https://doi.org/10.1074/jbc.M117.778308
9. Madhavi Sastry, G.; Adzhigirey, M.; Day, T.; Annabhimoju, R.; Sherman, W., Protein and ligand preparation: parameters, protocols, and influence on virtual screening enrichments. *Journal of Computer-Aided Molecular Design* **2013,** *27*(3), 221-234. https://doi.org/10.1007/s10822-013-9644-8
10. Protein Preparation Wizard; Epik, Schrödinger, LLC, New York, NY; Prime, Impact, Schrödinger, LLC, New York, NY, 2023. https://www.schrodinger.com/life-science/download/release-notes/release-2023-1/.
11. Eldridge, M. D.; Murray, C. W.; Auton, T. R.; Paolini, G. V.; Mee, R. P., Empirical scoring functions: I. The development of a fast empirical scoring function to estimate the binding affinity of ligands in receptor complexes. *J. Comput.-Aided Mol. Des.* **1997,** *11*(5), 425-445. https://doi.org/10.1023/A:1007996124545
12. Friesner, R. A.; Murphy, R. B.; Repasky, M. P.; Frye, L. L.; Greenwood, J. R.; Halgren, T. A.; Sanschagrin, P. C.; Mainz, D. T., Extra Precision Glide: Docking and Scoring Incorporating a Model of Hydrophobic Enclosure for Protein−Ligand Complexes. *J. Med. Chem.* **2006,** *49*(21), 6177-6196. https://doi.org/10.1021/jm051256o

13. Nandi, A.; Auti, P. S.; Jagtap, U. A.; Paul, A. T., Investigating the role of indole and quinazolinone-based hybrid analogues with ketoamide fragment and alkyl extension for potential PL inhibition. *J. Mol. Struct.* **2024,** *1301*, 137337. https://doi.org/10.1016/j.molstruc.2023.137337
14. Chattaraj, B.; Khanal, P.; Nandi, A.; Das, A.; Sharma, A.; Mitra, S.; Dey, Y. N., Network pharmacology and molecular modelling study of Enhydra fluctuans for the prediction of the molecular mechanisms involved in the amelioration of nephrolithiasis. *J. Biomol. Struct. Dyn.* **2023,** *41*(24), 15400-15410. https://doi.org/10.1080/07391102.2023.2189476
15. Jacobson, M. P.; Pincus, D. L.; Rapp, C. S.; Day, T. J. F.; Honig, B.; Shaw, D. E.; Friesner, R. A., A hierarchical approach to all-atom protein loop prediction. *Proteins: Struct., Funct., Bioinf.* **2004,** *55*(2), 351-367. https://doi.org/10.1002/prot.10613
16. Shelley, J. C.; Cholleti, A.; Frye, L. L.; Greenwood, J. R.; Timlin, M. R.; Uchimaya, M., Epik: a software program for pKaprediction and protonation state generation for drug-like molecules. *J. Comput.-Aided Mol. Des.* **2007,** *21*(12), 681-691. https://doi.org/10.1007/s10822-007-9133-z
17. Kongsted, J.; Ryde, U., An improved method to predict the entropy term with the MM/PBSA approach. *J. Comput.-Aided Mol. Des.* **2009,** *23*(2), 63-71. https://doi.org/10.1007/s10822-008-9238-z
18. Nandi, A.; Das, A.; Dey, Y. N.; Roy, K. K. The Abundant Phytocannabinoids in Rheumatoid Arthritis: Therapeutic Targets and Molecular Processes Identified Using Integrated Bioinformatics and Network Pharmacology *Life* [Online], 2023, p. 700.
19. Bowers, K. J.; Chow, E.; Xu, H.; Dror, R. O.; Eastwood, M. P.; Gregersen, B. A.; Klepeis, J. L.; Kolossvary, I.; Moraes, M. A.; Sacerdoti, F. D. In *Scalable algorithms for molecular dynamics simulations on commodity clusters*, Proceedings of the 2006 ACM/IEEE Conference on Supercomputing, 2006; pp 84-es.
20. Genheden, S.; Ryde, U., The MM/PBSA and MM/GBSA methods to estimate ligand-binding affinities. *Expert Opin. Drug Discovery* **2015,** *10*(5), 449-461. https://doi.org/10.1517/17460441.2015.1032936
21. Chattaraj, B.; Nandi, A.; Das, A.; Baidya, A.; Mahata, S.; Chowdhury, A.; Mitra, S.; Roy, S.; Chakraborty, S.; Dey, Y. N., Enhydra fluctuans Lour. aqueous extract inhibited the growth of calcium phosphate crystals: An in vitro study. *Food Chemistry Advances* **2023,** *2*, 100287. https://doi.org/10.1016/j.focha.2023.100287
22. Dixon, S. L.; Smondyrev, A. M.; Knoll, E. H.; Rao, S. N.; Shaw, D. E.; Friesner, R. A., PHASE: a new engine for pharmacophore perception, 3D QSAR model development, and 3D database screening: 1. Methodology and preliminary results. *J. Comput.-Aided Mol. Des.* **2006,** *20*(10), 647-671. https://doi.org/10.1007/s10822-006-9087-6
23. Nandi, A.; Nigar, T.; Das, A.; Dey, Y. N., Network pharmacology analysis of Plumbago zeylanica to identify the therapeutic targets and molecular mechanisms involved in ameliorating hemorrhoids. *J. Biomol. Struct. Dyn.*, 1-15. https://doi.org/10.1080/07391102.2023.2280681
24. Palakurti, R.; Sriram, D.; Yogeeswari, P.; Vadrevu, R., Multiple e-Pharmacophore Modeling Combined with High-Throughput Virtual Screening and Docking to Identify Potential Inhibitors of β-Secretase(BACE1). *Mol. Inf.* **2013,** *32*(4), 385-398. https://doi.org/10.1002/minf.201200169
25. Dixon, S. L.; Duan, J.; Smith, E.; Von Bargen, C. D.; Sherman, W.; Repasky, M. P., Autoqsar: An Automated Machine Learning Tool for Best-Practice Quantitative Structure–Activity Relationship Modeling. *Future Med. Chem.* **2016,** *8*(15), 1825-1839. https://doi.org/10.4155/fmc-2016-0093
26. Fan, N.; Zhang, S.; Sheng, T.; Zhao, L.; Liu, Z.; Liu, J.; Wang, X., Docking field-based QSAR and pharmacophore studies on the substituted pyrimidine derivatives targeting

HIV-1 reverse transcriptase. *Chem. Biol. Drug Des.* **2018,** *91*(2), 398-407. https://doi.org/10.1111/cbdd.13086
27. . https://www.schrodinger.com/products/qikprop
28. Daina, A.; Michielin, O.; Zoete, V., SwissADME: a free web tool to evaluate pharmacokinetics, drug-likeness and medicinal chemistry friendliness of small molecules. *Scientific Reports* **2017,** *7*(1), 42717. https://doi.org/10.1038/srep42717
29. Arijit, N.; Nandita, M.; Anwesha, D.; Yadu Nandan, D., Approaches Based on Network Pharmacology and Molecular Docking to Predict the Molecular Mechanism of Plumbago zeylanica's Anti-Inflammatory Action. *J. Biol. Regul. Homeostatic Agents* **2024,** *38*(3), 2055-2067. https://doi.org/10.23812/j.biol.regul.homeost.agents.20243803.161
